# Entropy-Driven 1D Magnetic Carbon Fibers Embedded into 3D Aerogel Enable Broadband Electromagnetic Wave Absorption

**DOI:** 10.1007/s40820-026-02212-w

**Published:** 2026-05-07

**Authors:** Tingting Zhao, Shiping Shao, Ke Bi, Yunxiang Tang, Lili Wu, Jiurong Liu, Zhou Wang, Fenglong Wang

**Affiliations:** 1 State Key Laboratory of Coatings for Advanced Equipment, Shandong University, Jinan, 250061 People’s Republic of China; 2 School of Materials Science and Engineering, Shandong University, Jinan, 250061 People’s Republic of China; 3 State Key Laboratory of Information Photonics and Optical Communications, School of Science, Beijing University of Posts and Telecommunications, Beijing, 100876 People’s Republic of China

**Keywords:** Electromagnetic wave absorption, Electrospinning, Carbon fibers, Carbon aerogel, Spinel ferrites

## Abstract

Traditional powder-type electromagnetic absorbers have become inadequate against the intensified radiation across broader frequency ranges, induced by the proliferation of intelligent communication devices. To address this, we present an innovation strategy to assemble spinel ferrites/carbon fibers into a carbon aerogel fabricated via carbonization of a graphene oxide (GO)/2,2,6,6-tetramethylpiperidine-1-oxyl (TEMPO)-oxidized cellulose nanofibers (TOCNF) composite aerogel. In the preparation, an entropy-driven design integrated with electrospinning was employed to synthesize the magnetic carbon fibers (MCFs), fully leveraging the complementary advantages of magnetic and carbon materials for optimal impedance matching and attenuation. As a result, the optimized magnetic carbon aerogel (MCA-2), composed of TOCNF, GO, and MCFs, exhibits exceptional broadband electromagnetic wave absorption, with multiple effective reflection loss (RL) peaks (RL ≤ − 10 dB) across the frequency range of 4.8–18 GHz, a minimum RL value of − 54.11 dB, and a maximum effective absorption bandwidth of 7.2 GHz. Additionally, the lightweight MCA-2 aerogel has excellent thermal insulation performance and excellent photothermal performance, which gives it broad application prospects in diverse and harsh environments.

**Supplementary Information:**

The online version contains supplementary material available at 10.1007/s40820-026-02212-w.

## Highlights

- Entropy-driven design is utilized to enhance the electromagnetic loss synergy mechanism.
- The combination of 1D fibers and 3D aerogel leverages their complementary advantages.
- The optimized magnetic carbon aerogel is characterized by strong attenuation and broadband absorption.

**Supplementary Information:**

The online version contains supplementary material available at 10.1007/s40820-026-02212-w.

## Graphical abstract

**Figure:**
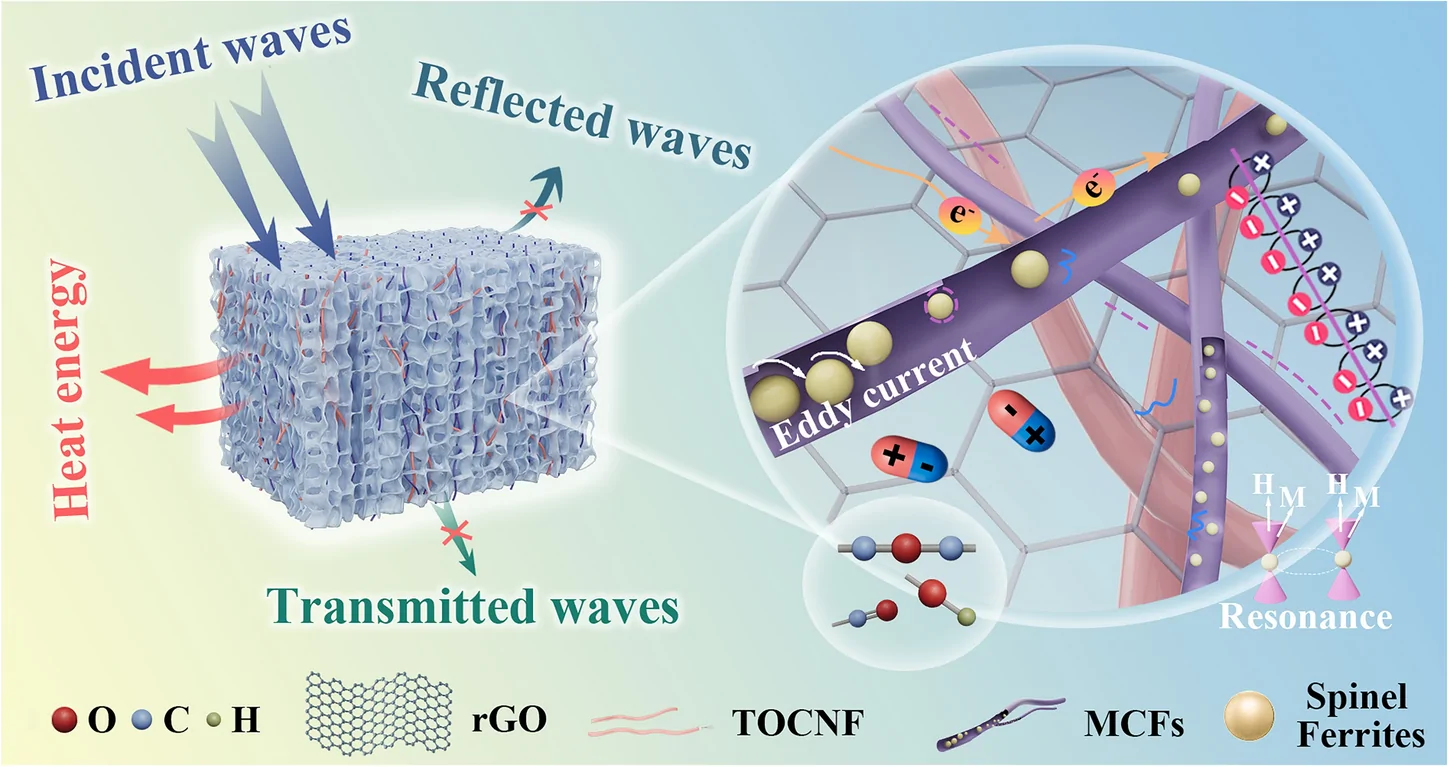

## Introduction

The increasing complexity of the electromagnetic environment, driven by the widespread use of highly informatized and intelligent electronic devices, has imposed stringent demands on electromagnetic wave (EMW) absorption materials [1–3]. Traditional powder-type absorbers such as magnetic powders (e.g., iron, cobalt, nickel metals, and their oxides) and non-magnetic carbon-based powders, hold a significant position in the field of EMW absorption due to their strong attenuation capabilities and low-cost advantages. However, despite the high reflection loss (RL) values (exceeding − 20 dB, corresponding to > 99% absorptivity of incident EMWs) reported in most studies, their effective absorption bandwidth (EAB) is typically limited to 4–6 GHz, demonstrating unsatisfactory results [4–10]. Therefore, developing a simple yet effective strategy to prepare broadband EMW-absorbing materials by fully leveraging the inherent advantages of traditional powders—such as their ease of dispersion in resin matrices, polymer emulsions, or integration into structural layers—represents a promising approach to address the challenges posed by the increasingly complex electromagnetic environment [11–14].

Considering that a reasonable combination of permeability and permittivity can effectively balance a material’s electromagnetic attenuation ability and impedance matching, the design of powdered micro-/nanohybrids through integrating magnetic and dielectric materials (particularly carbon-based materials, for instance, carbon fibers, carbon nanotubes, graphene, and biomass-derived carbon materials) has emerged as one of the mainstreams of high-efficiency EMW-absorbing materials [15–17]. Additionally, with the large-scale adoption of technologies such as electrospinning, freeze-drying, and three-dimensional (3D) printing in the composites’ manufacturing process, the microstructural design of broadband EMW-absorbing materials has exhibited a trend toward increasing diversity [18–24]. Among carbon materials, one-dimensional (1D) magnetic carbon nanofibers fabricated via electrospinning offer excellent compositional and structural designability. The in situ growth of magnetic nanoparticles within the carbon fibers provides abundant heterogeneous interfaces for EMW scattering and loss. However, the performance of these magnetic carbon nanofibers is fundamentally limited by the electromagnetic properties of the incorporated magnetic nanoparticles [25–28]. Conventional spinel ferrites (MFe_2_O_4_, where M=Fe, Co, Ni, Mn, Zn, etc.), while widely used, often suffer from a narrow range of tunable magnetic properties. This limitation stems from their fixed cation stoichiometry, which constrains the degrees of freedom for modulating ferromagnetic resonance frequency, ultimately hindering the simultaneous optimization of impedance matching and broadband electromagnetic loss. To break this limitation and achieve unprecedented tunability in electromagnetic properties, entropy-driven engineering has emerged as a revolutionary materials design strategy. By introducing multiple cations into a single lattice site, entropy-design spinel ferrites exhibit unique microstructures and enhanced properties, such as improved saturation magnetization, higher magnetic loss, and greater flexibility in tailoring both permittivity and permeability. These enhancements arise from the severe lattice distortion, cocktail effect, and sluggish diffusion kinetics [29–31]. Therefore, integrating entropy-driven spinel ferrites into 1D carbon nanofibers represents a promising avenue to create highly efficient and tunable EMW-absorbing units.

In addition to magnetic carbon nanofibers, carbon aerogels inlaid with electromagnetic functional powders also have similar advantages. But unlike fibers, aerogels typically exhibit a 3D porous structure with a large specific surface area. The high air content within the material enables closer impedance matching with free space. The large surface area provides numerous sites for particle loading, while the long and tortuous pore channels prolong the propagation path of EMWs, thereby promoting their scattering and attenuation within the material [32–35]. However, standalone 1D fibers may suffer from aggregation and limited structural complexity, while pure 3D aerogels might lack sufficient magnetic loss centers. Accordingly, the rational integration of 1D functional nanofibers into a 3D conductive network through freeze-assisted self-assembly offers a promising solution [36, 37]. Unlike conventional aerogel-based composites, this multi-dimensional integration strategy simultaneously introduces magnetic loss and finely modulates the formation of the three-dimensional conductive network. This dual modulation optimizes impedance matching and enhances attenuation capability, thereby facilitating the development of high-performance EMW absorbers featured with broad bandwidth and lightweight properties.

Herein, magnetic carbon fibers (MCFs) decorated with in situ grown spinel ferrite particles ((Mn_0.05_Ni_0.45_Zn_0.05_Co_0.45_)Fe_2_O_4_) engineered through an entropy-driven design with compositions optimized via preliminary experiments. These MCFs were then embedded into a graphene oxide/TEMPO-oxidized cellulose nanofibers (TOCNF) framework. Subsequently, an appropriate annealing process was applied to produce a magnetic carbon aerogel with impressive broadband EMW absorption performance. Since the dielectric difference between carbon aerogel and MCFs helps mitigate the impedance mismatch induced by the 3D conductive framework of carbon aerogel, the composite aerogel’s strong attenuation ability, which originates from diverse electromagnetic losses, allows it to effectively dissipate more EMWs that penetrate deep into the material. As a result, MCA-2 aerogel exhibits an extensive maximum EAB of 7.2 GHz, and its minimum RL reaches − 54.11 dB with corresponding matching thickness of 2.02 mm. Moreover, the effective RL peaks (RL ≤ − 10 dB) at different thicknesses within 1–5 mm show an average value of − 26.77 dB and fall into a wide range from C to Ku bands (4.8–18 GHz), demonstrating a fairly good EMW absorption performance. Moreover, the lightweight and flexible hybrid aerogel also exhibits excellent thermal insulation and efficient photothermal conversion capabilities, demonstrating broad application potential across various practical scenarios. This work highlights the great potential of integrating entropy-engineered functional fillers into sophisticated hierarchical architectures, offering a powerful strategy for developing next-generation EMW-shielding materials. Furthermore, this work provides valuable guidance for enhancing the broadband EMW absorption performance of traditional powdered absorbers through rational compositional design and tailored structural architectures.

## Experimental Section

### Materials

Manganese (II) acetylacetonate (Mn(acac)_2_, AR), Nickel(II) acetylacetonate (Ni(acac)_2_, AR), Zinc(II) acetylacetonate (Zn(acac)_2_, AR), Cobalt(II) acetylacetonate (Co(acac)_2_, AR), and Iron(III) acetylacetonate (Fe(acac)_3_) were bought from Shanghai Aladdin Biochemical Technology Co., Ltd. (China). Polyacrylonitrile (PAN, *M*_w_ = 250,000) was purchased from Shanghai Macklin Biochemical Technology Co., Ltd. (China). N, N-Dimethylformamide (DMF, AR) was bought from Sinopharm Chemical Reagent Co., Ltd. (China). Graphene oxide (GO) and TEMPO-oxidized cellulose nanofibers (TOCNF) were supplied by Hangzhou TanFeng Technology Co., Ltd. (China) and Tianjin Mujingling Biochemical Technology Co., Ltd. (China), respectively. All chemical reagents were directly used without further purification.

### Synthesis of MCFs

The detailed synthetic procedures of MCFs are presented in Section S1 in Supporting Information.

### Fabrication of Magnetic Carbon Aerogels

In total, 200 mg of GO powders and 800 mg of TOCNF were sequentially dispersed in 20 mL of deionized water by stirring and ultrasonication. Subsequently, a certain mass of the previously prepared MCFs was added to the aforesaid mixed solution. After thorough stirring and ultrasonic oscillation, the resulting solution was poured into a square-shaped Teflon mold with a copper base. The TOCNF/GO/MCFs composite aerogel was then obtained through rapid freezing in liquid nitrogen followed by a vacuum freeze-drying process (− 55 °C, < 10 Pa) after 48 h. Finally, the obtained composite aerogel was transferred to a tube furnace and annealed at 300 °C for 3 h under a nitrogen atmosphere (flow rate: 100 mL min^−1^) with a heating rate of 5 °C min^−1^, followed by natural cooling to room temperature. According to the mass of MCFs incorporated into aerogel, the samples were named as CA (0 mg), MCA-1 (2.5 mg), MCA-2 (5 mg), MCA-3 (10 mg), respectively.

### Characterization

The morphology and microstructure of MCFs and MCAs were observed by using a field-emission scanning electron microscope (FE-SEM, JEOL-7610F). All HR-TEM images and SAED pattern were obtained through a high-resolution transmission electron microscopy (HR-TEM; JEM-2100). The X-ray diffractometer with Cu Kα radiation (XRD, DMAX-2500 PC) was applied to analyze the crystal structure of MCFs and the phase composition of MCAs over the 2θ range of 10–90°. The carbon graphitization degree and defect density of MCFs and MCAs were studied via a Raman spectrometer (LabRAM HR evolution). The X-ray photoelectron spectroscopy (XPS, Thermo ESCALAB 250Xi) and Fourier transform infrared spectrometer (Nicolet iS 5 FT-IR) were used to compare the surface chemical states and changes in functional groups of composite aerogels before and after carbonization. The thermal insulation and photothermal performance of aerogels was assessed by a thermal infrared imager (FLIR E86). A Dynamic Mechanical Analyzer (DMA, TA Q800) was employed to evaluate the mechanical properties. Magnetic properties of MCFs and MCAs were explored by vibration sample magnetometer (VSM, LakeShore-7404) at room temperature. As for electromagnetic measurements, the detailed measurement procedure is provided in Section S1. In addition, all corresponding calculation formulas for EMW absorption performance are given in Section S3.

## Results and Discussion

### Composition and Structure

**Fig. 1:**
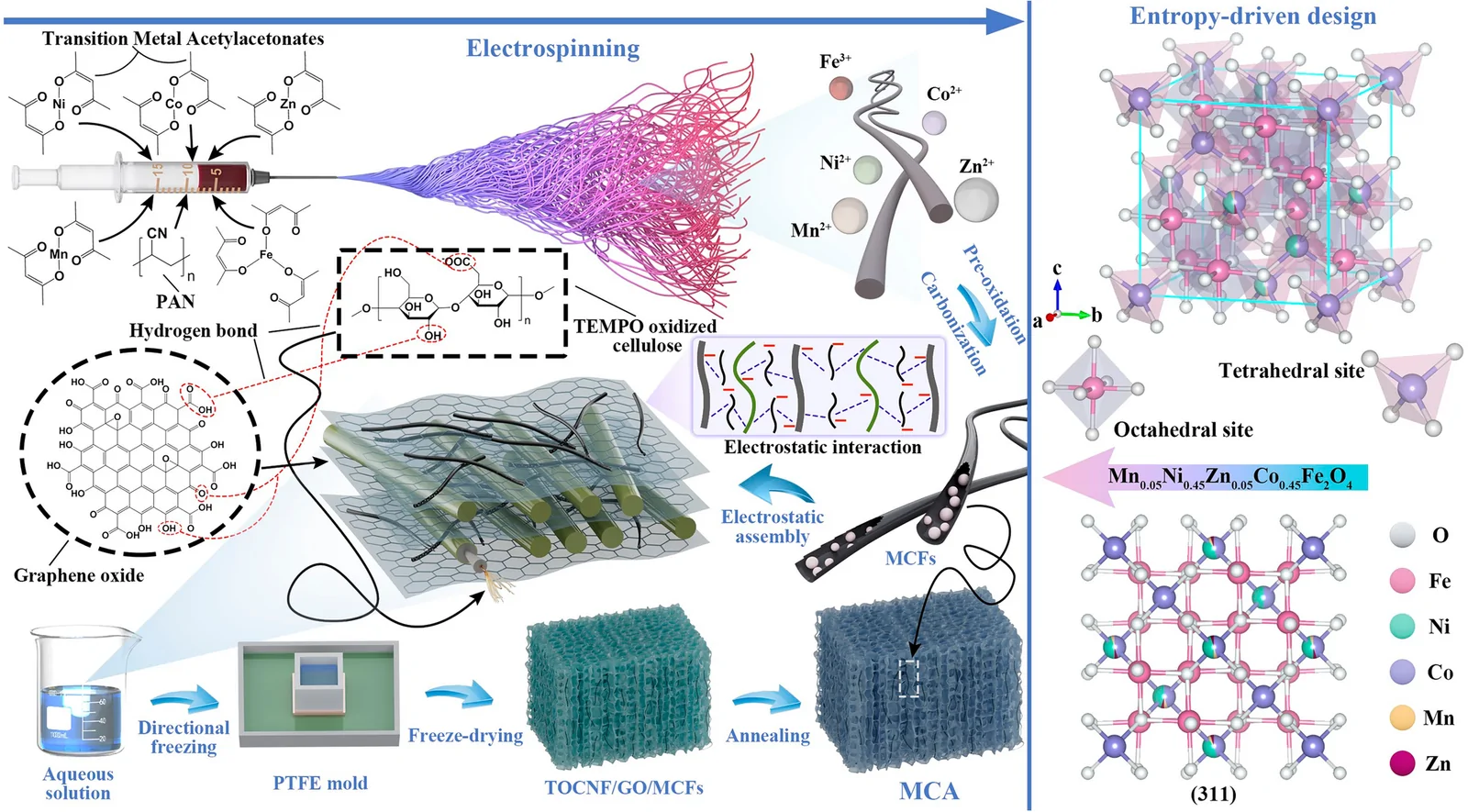
Schematic diagram for the preparation of MCFs and MCA

MCFs and MCA hybrid aerogels were prepared via the electrospinning and freeze-drying process, respectively. As clearly shown in Fig. 1, entropy-driven design of spinel ferrites can be readily achieved by introducing different metal ions to the precursor solution. During the pre-oxidation and carbonization process, the in situ growth of (Mn_0.05_Ni_0.45_Zn_0.05_Co_0.45_) Fe_2_O_4_ spinel ferrites (*S*_config_ = 0.65R, calculated by Eq. S1) was gradually completed, concomitant with the slow cyclization and dehydrogenation of PAN [30]. This resulted in MCFs possessing excellent magnetic and electrical properties, thus endowing the fibers with multiple electromagnetic loss mechanisms. The similar surface charges of GO nanosheets, TOCNF, and MCFs facilitated them to be evenly dispersed in deionized water via electrostatic interaction (Fig. S1). More importantly, the functional groups (–OH, –COOH) of GO and TOCNF tended to form hydrogen bonds, thereby promoting the formation of a stable skeleton after freeze-drying. The advancing fronts of rapidly growing ice crystals are conducive to the formation of pore walls in aerogel through repelling the mixtures to accumulate them on both sides. After the ice crystals sublimated, they left behind numerous air-filled pores in their original location [38, 39]. Subsequent annealing treatment substantially reduced oxygen-containing functional groups and partially carbonized the cellulose. This process ultimately constructed a conductive carbon aerogel embedded with MCFs. By integrating 1D MCFs with a 3D carbon aerogel, this multi-dimensional design enables a synergistic effect that leads to superior performance in broadband EMW absorption.

**Fig. 2:**
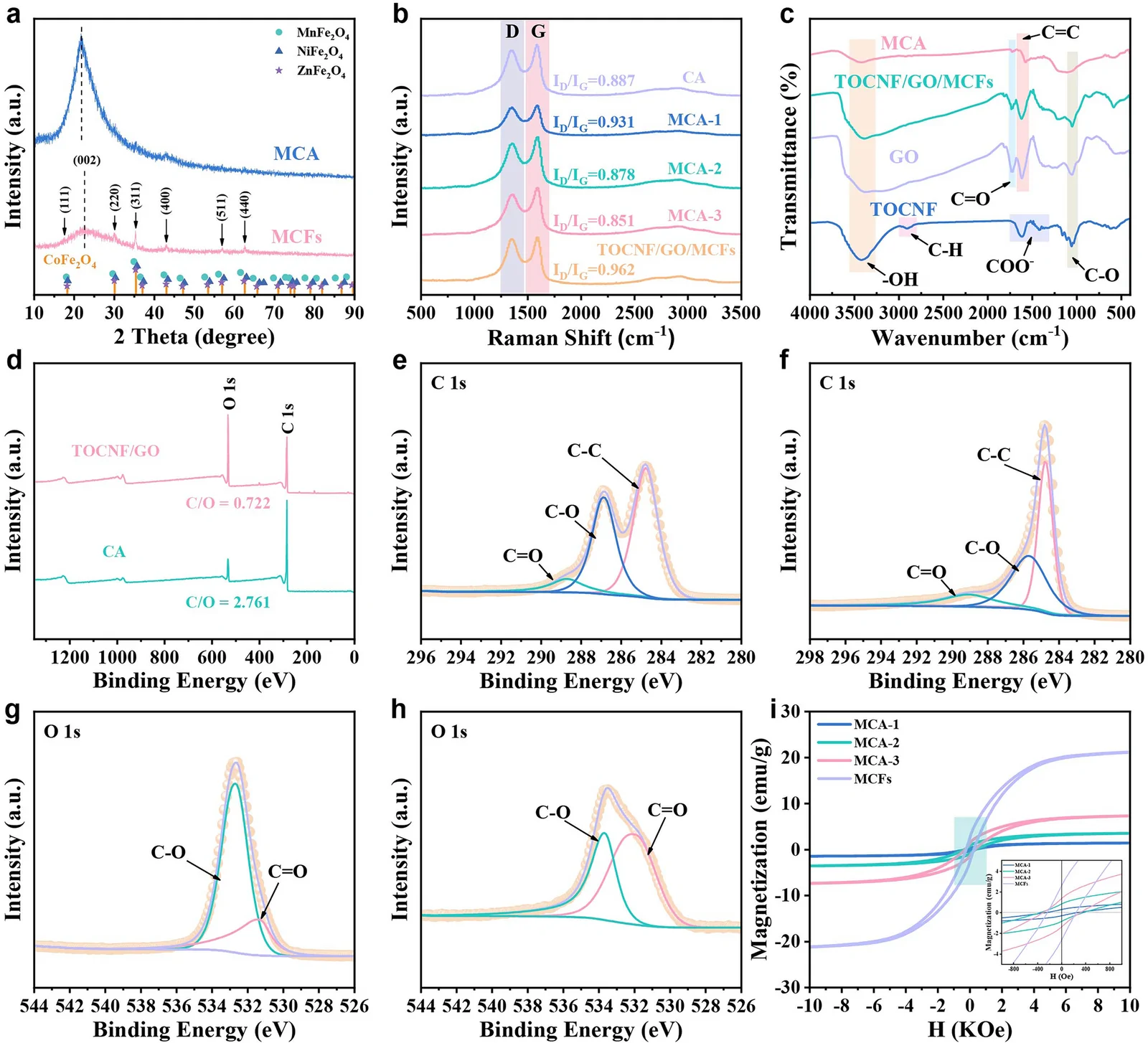
**a** XRD patterns of MCFs and MCA. **b** Raman spectra of CA, MCA-1, MCA-2, MCA-3, and TOCNF/GO/MCFs composite aerogels. **c** FT-IR spectra of GO, TOCNF, TOCNF/GO/MCFs composite aerogel and MCA. **d** XPS survey scan spectra of CA before and after carbonization. C 1*s* high-resolution XPS spectra of **e** TOCNF/GO aerogel and **f** CA. O 1*s* high-resolution XPS spectra of **g** TOCNF/GO aerogel and **h** CA. **i** Hysteresis loops of MCFs and MCA samples

To identify the crystal structure and phase composition of the MCFs and MCA, the XRD patterns of both MCFs and MCA were analyzed. As exhibited in Fig. 2a, several typical peaks corresponding solely to spinel ferrites (MFe_2_O_4_, M = Mn, Ni, Zn, Co) are observed, thus preliminarily proving the success of entropy-driven design. The obvious diffraction peaks around 30°, 35.2°, 43°, 57°, 62.6°, along with a faint peak at 17.5°, are indexed to (220), (311), (400), (511), (440), and (111) crystal planes, respectively [40, 41]. The wide characteristic peak observed at 22.4° can be assigned to the (002) crystal plane of graphite. For MCA, the presence of a strong and broad diffraction peak at 21.7°, corresponding to the (002) plane of graphite, reveals the successful formation of a carbon skeleton. The graphitization degree and defect density of the carbon skeleton were studied through Raman spectra. As shown in Fig. 2b, all samples exhibit two characteristic peaks, the D-band near 1350 cm^−1^ and the G-band near 1580 cm^−1^. The D-band is associated with disordered carbon structures or defects, while the G-band corresponds to graphitic carbon (*sp*^2^-hybridized carbon). The intensity ratio of the D-band to G-band (I_D_/I_G_) is a widely accepted metric for quantifying the defect density within carbon materials. Before annealing, the TOCNF/GO/MCFs composite aerogel exhibits an I_D_/I_G_ ratio of 0.962. As evidenced by the decreased I_D_/I_G_ ratio, the annealing process, which involves the reduction of GO and carbonization of TOCNF, improves the structural ordering by enhancing graphitization and minimizing defects in the carbon skeleton. Interestingly, the I_D_/I_G_ ratio exhibited a non-monotonic trend with increasing MCFs content, suggesting a possible dual competitive mechanism affecting the ordering of the carbon skeleton. At low loadings, MCFs may act as defect sources, where heterogeneous interfaces introduce disorder. At high loadings, they may function as graphitization templates, inducing the ordered arrangement of the surrounding amorphous carbon matrix. While defects can disrupt the continuous conductive network, thereby impairing conductive loss, they are not always detrimental to EMW-absorbing materials. Defect sites can contribute to EMW attenuation by enhancing dipole polarization. Changes in oxygen-containing functional groups of carbon aerogel indirectly reflect the conversion of TOCNF/GO to carbon skeleton. Figure 2c shows the FT-IR spectra of MCA and TOCNF/GO/MCFs composite aerogel. Four characteristic peaks are observed at 3421, 1720, 1618, and 1054 cm^−1^, corresponding to the stretching vibration of –OH, C=O, C=C, and C–O, respectively [42, 43]. Compared to other samples, the reduction in intensity of the corresponding peaks indicates the substantial removal of oxygen-containing functional groups and the restoration of the *sp*^2^ carbon network. This can be further confirmed by a significant rise in C/O ratio (Fig. 2d) and the changes in C–C, C–O, and C=O bonds observed in the C 1*s* and O 1*s* high-resolution XPS spectra (Fig. 2e–h). To evaluate the influence of MCFs on carbon aerogels’ magnetic properties, the saturation magnetization (*M*_s_) and coercivity (*H*_c_) of the samples were compared based on their hysteresis loops (Fig. 2i). The *M*_s_ of MCA increases with the addition amount of MCFs. Specifically, the *M*_s_ values are 1.47, 3.71, 7.62, and 21.88 emu g^−1^ for MCA-1, MCA-2, MCA-3, and MCFs, respectively. The *H*_c_ values of MCA-1, MCA-2, MCA-3, and MCFs are 261.7, 362.3, 377.1, and 242.2 Oe, respectively. The gradually enhanced coercivity leads to a shift of the optimal RL peak toward higher frequency, which is consistent with the ferromagnetic resonance theory (Eqs. S2–S4) [44, 45]. Even if a low *M*_s_ means MCFs cannot provide sufficient magnetic loss like pure magnetic metals, it still can play a crucial role in regulating the electromagnetic parameters. This regulation ultimately balances the impedance matching between MCA and free space. Density functional theory (DFT) simulations further reveal that entropy-driven multi-metal doping induces overlapping d-orbitals near the Fermi level, broadening the conduction band and enhancing electron delocalization (Fig. S2). This improved electronic structure facilitates better coupling between dielectric and magnetic loss, contributing to superior electromagnetic wave absorption. Additionally, the localized variation in electron density within the entropy-driven crystal structure may induce local electron resonance or enhance the electric field effect, thereby contributing to improved absorption performance [46–48].

**Fig. 3:**
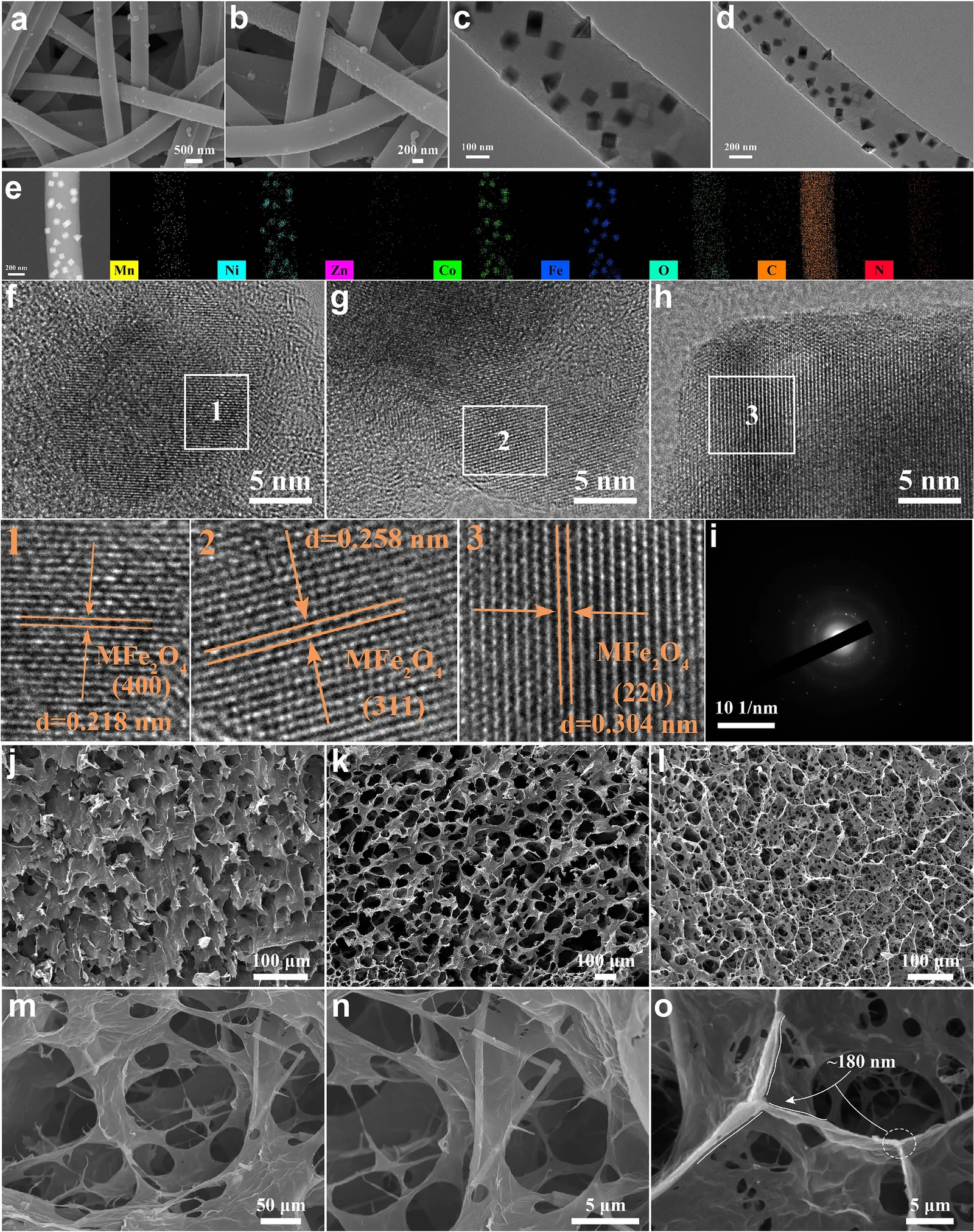
**a, b** SEM images of MCFs. **c, d** TEM images and **e** corresponding EDS mappings of MCFs. **f–h** HR-TEM images and **i** SAED pattern of MCFs. **j–o** SEM images of MCA

The microstructure and surface morphology of the MCFs and the MCA clearly reveal the multi-dimensional design. Entropy-driven designed spinel ferrites are scattered inside or on the surface of carbon fibers. The average diameter of carbon fibers is 450 nm, while the average size of particles reaches ~ 60 nm (Fig. 3a–d). EDS mappings (Fig. 3e) confirmed the existence of Mn, Ni, Zn, Co, and Fe elements originated from transition metal acetylacetonates, along with C, O, and N elements. The different mole ratios of metal ions in (Mn_0.05_Ni_0.45_Zn_0.05_Co_0.45_)Fe_2_O_4_ spinel ferrites caused the diversity in brightness of mappings. The difference in electronegativity enables the N heteroatoms within the graphite carbon network to promote the conductive loss of MCFs. Three types of crystal planes including (400), (311), and (220) can be found in HR-TEM images and SAED pattern (Fig. 3f–i), and their interplanar distances are 0.218, 0.258, and 0.304, respectively. Notably, the expansion of the aforementioned interplanar spacings reflects an increase in the structural disorder of the crystal, while also corroborating the successful implementation of the entropy-driven strategy. As for MCA, it exhibits a hierarchically porous structure induced by the typical freeze-drying process. Quantitative analysis via mercury intrusion porosimetry (MIP) further corroborates the hierarchical nature of this porous architecture (Fig. S3), yielding a porosity of approximately 88%. Combined with SEM analysis (Figs. 3j–l and S4a–d), the GO nanosheets and TOCNF align along the direction of ice crystal growth and intertwine to form a hierarchical structure, which features honeycomb-like macropores interlaced with smaller pores created by carbonized cellulose. Interestingly, this structure supports the aforementioned proposal that MCFs function as graphitization templates, which direct the ordered alignment of the adjacent amorphous carbon matrix. The uniform stacking of TOCNF on GO nanosheets significantly increases the thickness of the macropore walls, thereby providing stable support for the carbon aerogel. Meanwhile, the carbonized thin walls derived from MCFs as templates not only offer additional loading sites for MCFs but also enhance the overall flexibility of the carbon skeleton to some extent. The heterogeneous interfaces formed by the embedding or dispersion of MCFs within the carbon skeleton endow MCA with profuse interfacial polarization (Figs. 3m–o and S4e, f). Acting as loss units, these interfaces synergize with the carbon skeleton to effectively attenuate incident EMWs through multiple electromagnetic loss mechanisms. The low-magnification SEM image (Fig. S4g) reveals a well-preserved porous network, and corresponding EDS mappings show that the transition metal elements (Fe, Co, Ni, Mn, Zn) are uniformly distributed, confirming the homogeneous incorporation of MCFs. This uniform dispersion is critical for establishing an efficient conductive network and maximizing heterogeneous interface formation, both of which contribute to the enhanced electromagnetic wave absorption performance.

### Electromagnetic Properties and Mechanism

**Fig. 4:**
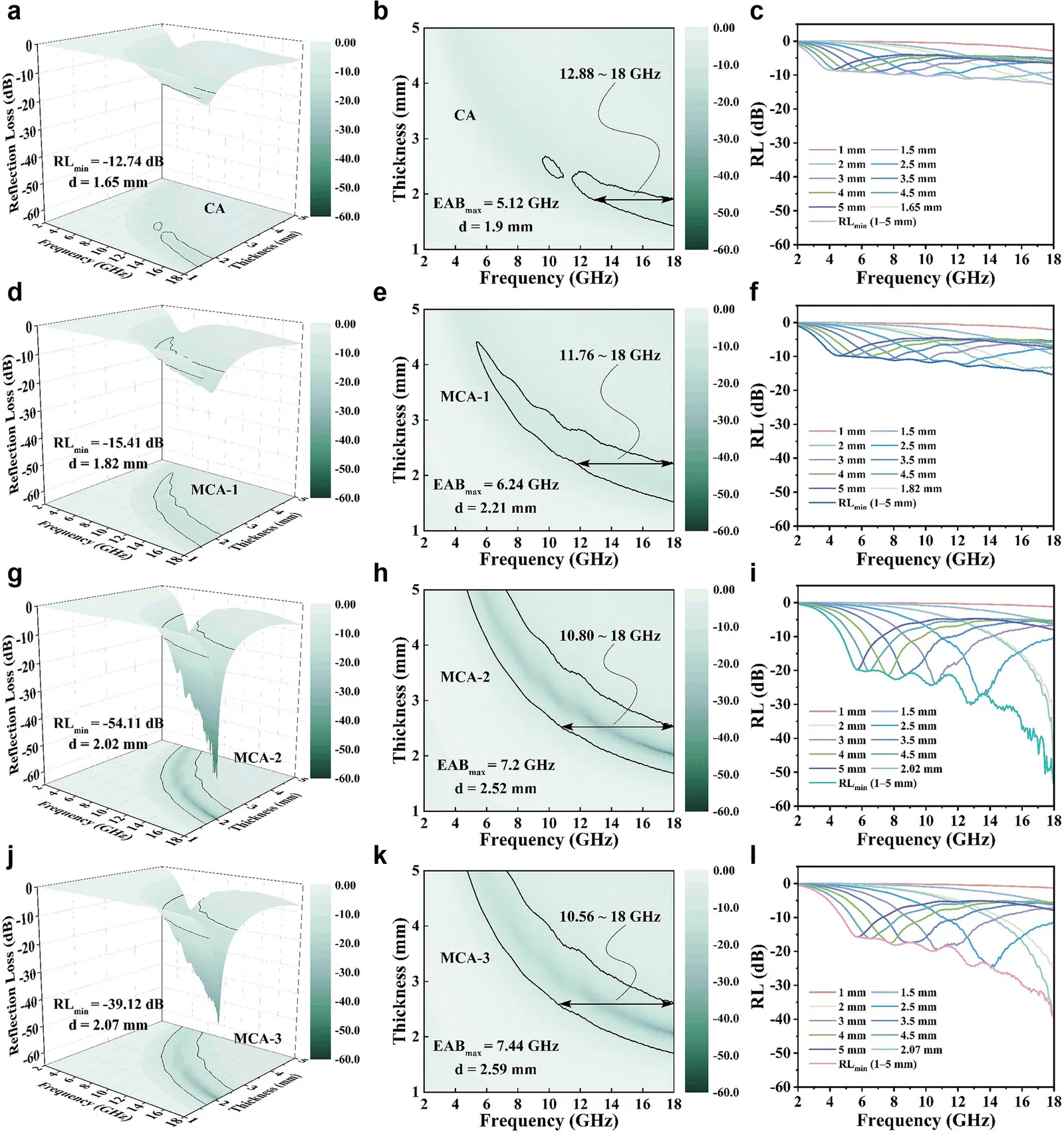
**a** 3D RL image, **b** corresponding 2D RL plot, and **c** RL-*f* curves at different thickness for CA. **d** 3D RL image, **e** corresponding 2D RL plot, and **f** RL-*f* curves at different thickness for MCA-1. **g** 3D RL image, **h** corresponding 2D RL plot, and **i** RL-*f* curves at different thickness for MCA-2. **j** 3D RL image, **k** corresponding 2D RL plot, and **l** RL-*f* curves at different thickness for MCA-3

To validate the effect of the multi-dimensional design on MCA’s EMW absorption performance, the EAB and RL values of the samples are presented in Fig. 4 (calculated by Eqs. S5 and S6). Compared to CA, the minimum RL values for MCA-1, MCA-2, and MCA-3 were recorded at − 15.41, − 54.11, and − 39.12 dB, respectively. The results represent a significant improvement over CA’s value of − 12.74 dB (Fig. 4a, d, g, and j). Correspondingly, their maximum EAB values increased to 6.24, 7.2, and 7.44 GHz from CA’s value of 5.12 GHz (Fig. 4b, e, h, and k). These results demonstrate that incorporating MCFs into the carbon aerogel substantially enhances overall EMW absorption performance, demonstrating the effectiveness of the multi-dimensional design strategy. The RL-*f* curves intuitively reflect the frequency range covered by the effective RL peaks (RL ≤ − 10 dB) across a thickness range of 1–5 mm (Fig. 4c, f, i, and l). Notably, the minimum RL values of MCA-2 and MCA-3 appear at 18 GHz and their maximum EAB covers the whole Ku band (12–18 GHz). Additionally, an average RL value of − 26.77 dB is achieved by MCA-2 over the frequency range of 4.2–18 GHz, highlighting its potential as a high-performance broadband EMW-absorbing material.

**Fig. 5:**
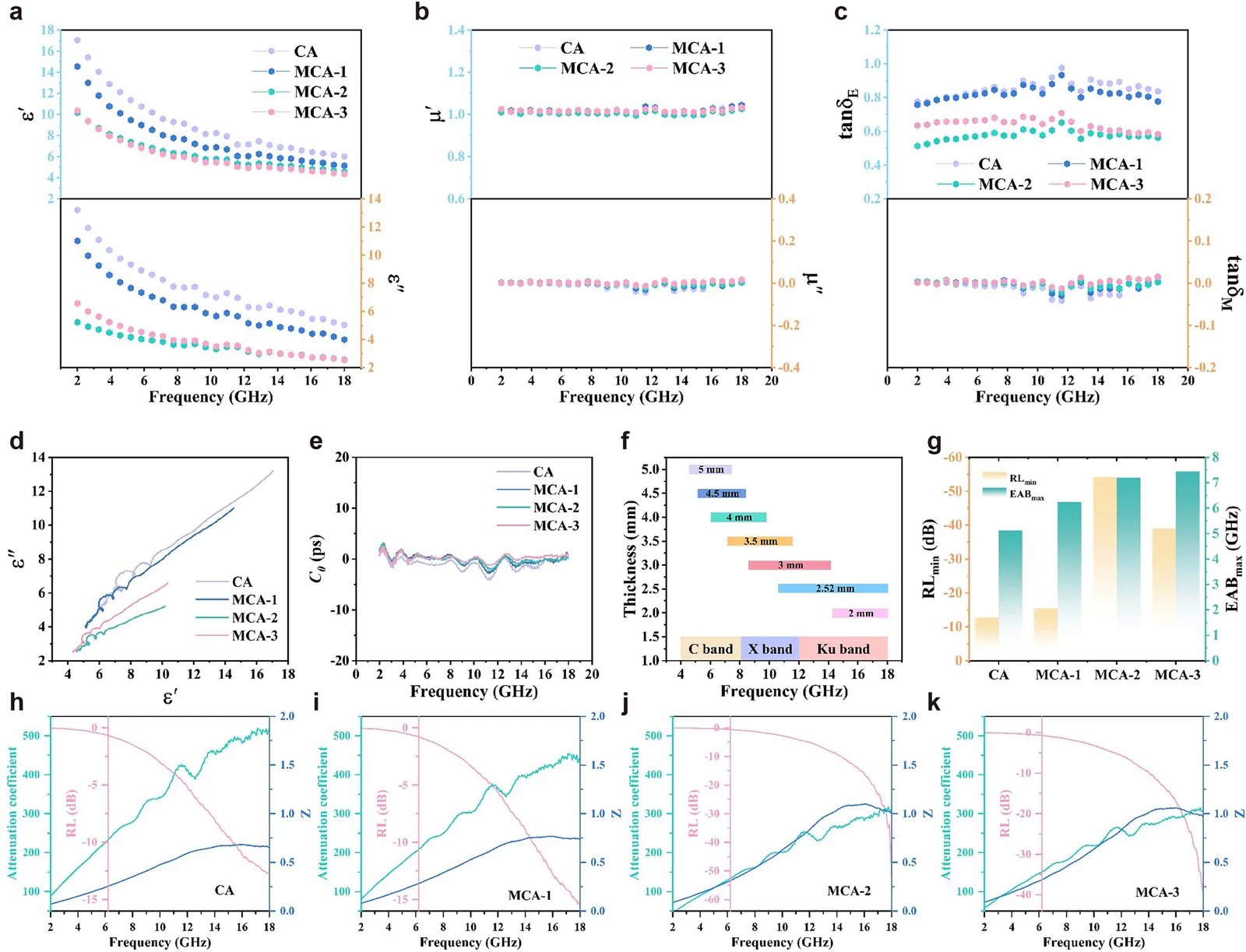
**a** Permittivity, **b** permeability, **c** dielectric and magnetic tangent loss, **d** Cole–Cole curves, and **e** *C*_0_*–f* curves of composite aerogels. **f** Maximum EAB of MCA-2 at different thickness. **g** Comparison of RL and EAB for prepared samples. Attenuation coefficient, RL–*f* and Z–*f* curves for **h** CA, **i** MCA-1, **j** MCA-2, and **k** MCA-3

To get an in-depth understanding on the impressive broadband EMW absorption performance of MCA, the complex permittivity (*ε*_r_ = *ε′ − jε″*) and the complex permeability (*μ*_r_ = *μ′ − jμ″*) of all samples were measured. As depicted in Fig. 5a, MCFs in the composite aerogel play a key role in modulating the permittivity of the carbon skeleton, which is highly susceptible to a severe impedance mismatch due to the skin effect induced by the high permittivity. In Fig. 5b, the permeability of the samples exhibits only minor fluctuations. This results in a non-trivial yet constrained contribution to the electromagnetic parameters compared to non-magnetic materials. Crucially, it avoids the impedance mismatch detrimental to EMW absorption, which is often induced by an excessively high permeability. Figure 5c indicates that dielectric loss plays a dominant role and contributes primarily to the attenuation of EMWs. The dielectric loss mechanisms can be further refined through the analysis of Cole–Cole curves (Figs. 5d and S5a). The Cole–Cole curve of the MCA-2 sample exhibits a shortened semicircular arc and a decreased slope in the linear region. According to Debye theory (Eqs. S7–S9), these characteristics suggest a more diverse set of relaxation processes and a broader distribution of relaxation times. This diversity originates from the synergistic effects of multiple loss mechanisms. Moreover, the wider distribution of relaxation times indicates that the material can effectively absorb EMWs over a broader frequency range. The magnetic loss mechanisms were investigated using *C*_0_*–f* curves (Figs. 5e and S5b), calculated by Eq. S10. The results reveal that natural resonance and exchange resonance, rather than eddy current loss, are the dominant magnetic loss mechanisms [49, 50]. The synergy between the dielectric and magnetic loss mechanisms endows the MCA-2 sample with excellent broadband EMW absorption performance, extending its EAB to the C band (Fig. 5f, g). Furthermore, compared to other samples, the intersection point of the curves for MCA-2 is closer to the peak attenuation constant (calculated by Eq. S11), the point where Z = 1 on the Z*–f* curve (calculated by Eq. S12), and the minimum RL. This implies that MCA-2 achieves a more optimal balance between impedance matching and attenuation capability (Fig. 5h–k), which is a key factor contributing to its excellent performance.

**Fig. 6:**
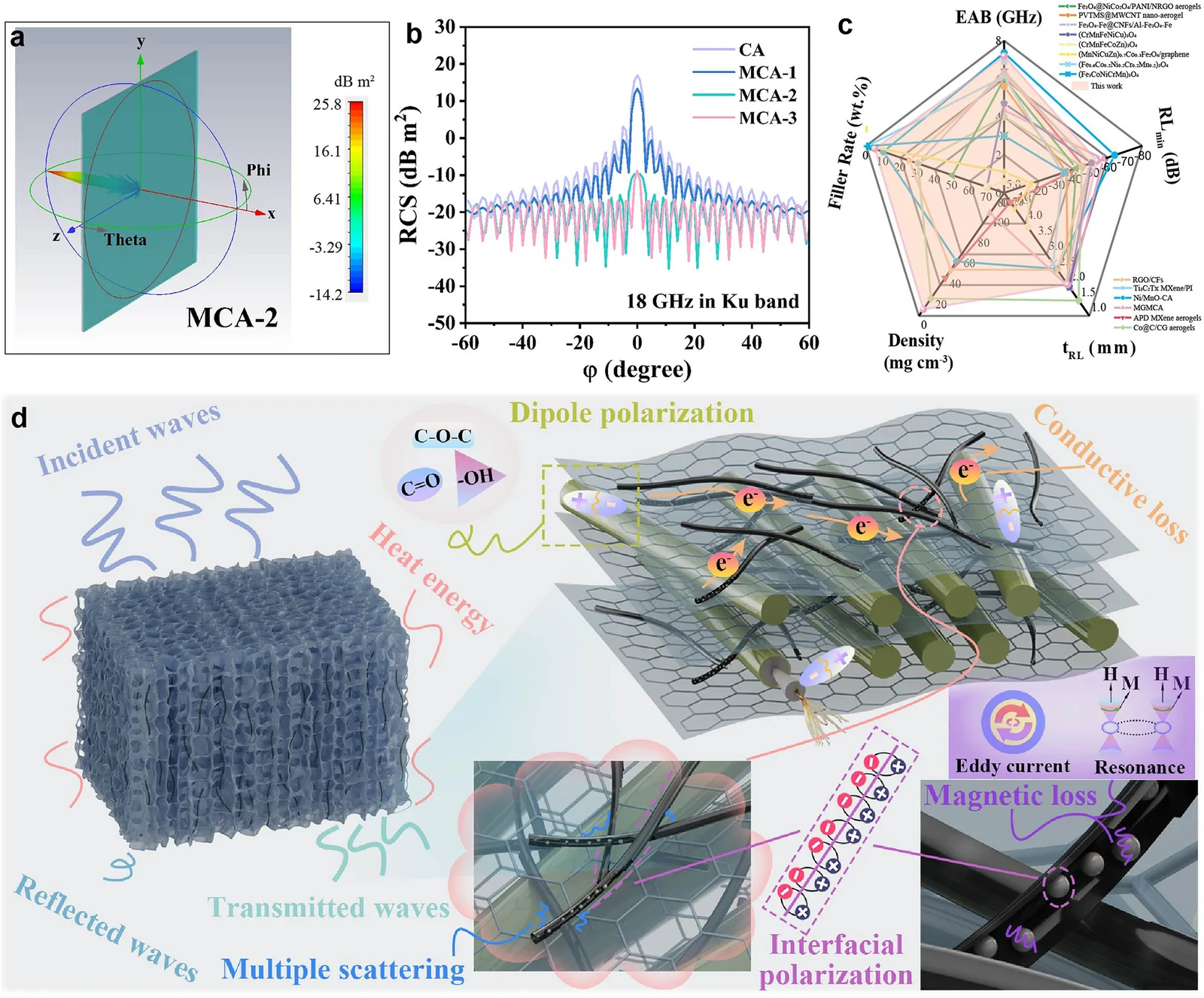
**a** 3D RCS diagrams of MCA-2. **b** 2D RCS values of samples. **c** Radar chart comparing the comprehensive performance of MCA-2 with recently reported electromagnetic wave absorbers. **d** Schematic illustration of the EMW absorption mechanism for MCA

To further evaluate the EMW absorption performance of MCA, the radar cross section (RCS) of the samples was simulated (detailed parameter settings are provided in Section S4). In the 3D RCS diagrams, compared to the distinct reflected waves observed on the metal backplate side, those on the simulated material side are significantly reduced or even disappear (Figs. 6a and S6). In addition, the maximum 2D RCS values (calculated by Eq. S13) of MCA-2 and MCA-3 are below − 10 dB m^2^, and their average values are below − 20 dB m^2^ (Fig. 6b). Figure S7a presents the RCS reduction values calculated as the difference between the coated sample and bare PEC. MCA-2 achieves a maximum RCS reduction value of 35.3 dB m^2^ at normal incidence and exhibits exceptional attenuation capabilities at observation angles such as 0°, 15°, and 45°. Besides, simulations were further extended to horizontal polarization. As shown in Fig. S7b, MCA-2 exhibits similarly excellent attenuation performance, with RCS curves displaying nearly identical trends across the entire angular range under both polarization conditions. The above results further demonstrate that the appropriate introduction of MCFs positively contributes to the EMW absorption performance of the carbon aerogel. As illustrated in the radar chart (Fig. 6c) and summarized in Table S1, MCA-2 exhibits outstanding comprehensive performance compared with previously reported materials, particularly in terms of low density, strong reflection loss, broad effective absorption bandwidth, and low filler loading [51–63]. Based on the preceding discussions, the EMW absorption mechanism of the MCA material was analyzed, and a corresponding schematic illustration is presented in Fig. 6d. The excellent EMW absorption performance of MCA can be attributed to the compositional and structural advantages arising from its multi-dimensional design. Specifically, MCFs in carbon aerogel can act as numerous attenuation units that dissipate incident EMWs primarily via dielectric loss, including conductive loss, interfacial polarization, and dipole polarization. As for carbon aerogel, it provides an ideal platform for MCFs, with strong conductive loss and a long, tortuous EMWs’ propagation path. Moreover, the MCFs not only form abundant heterogeneous interfaces with the carbon skeleton to enhance interfacial polarization but also balance the conductive network through the difference in permittivity and changes of permeability to optimize the impedance matching between the material and free space. These features make MCA a promising candidate for highly efficient EMW-absorbing materials with a wide EAB.

### Mechanical Performance and Thermal Management Performance

**Fig. 7:**
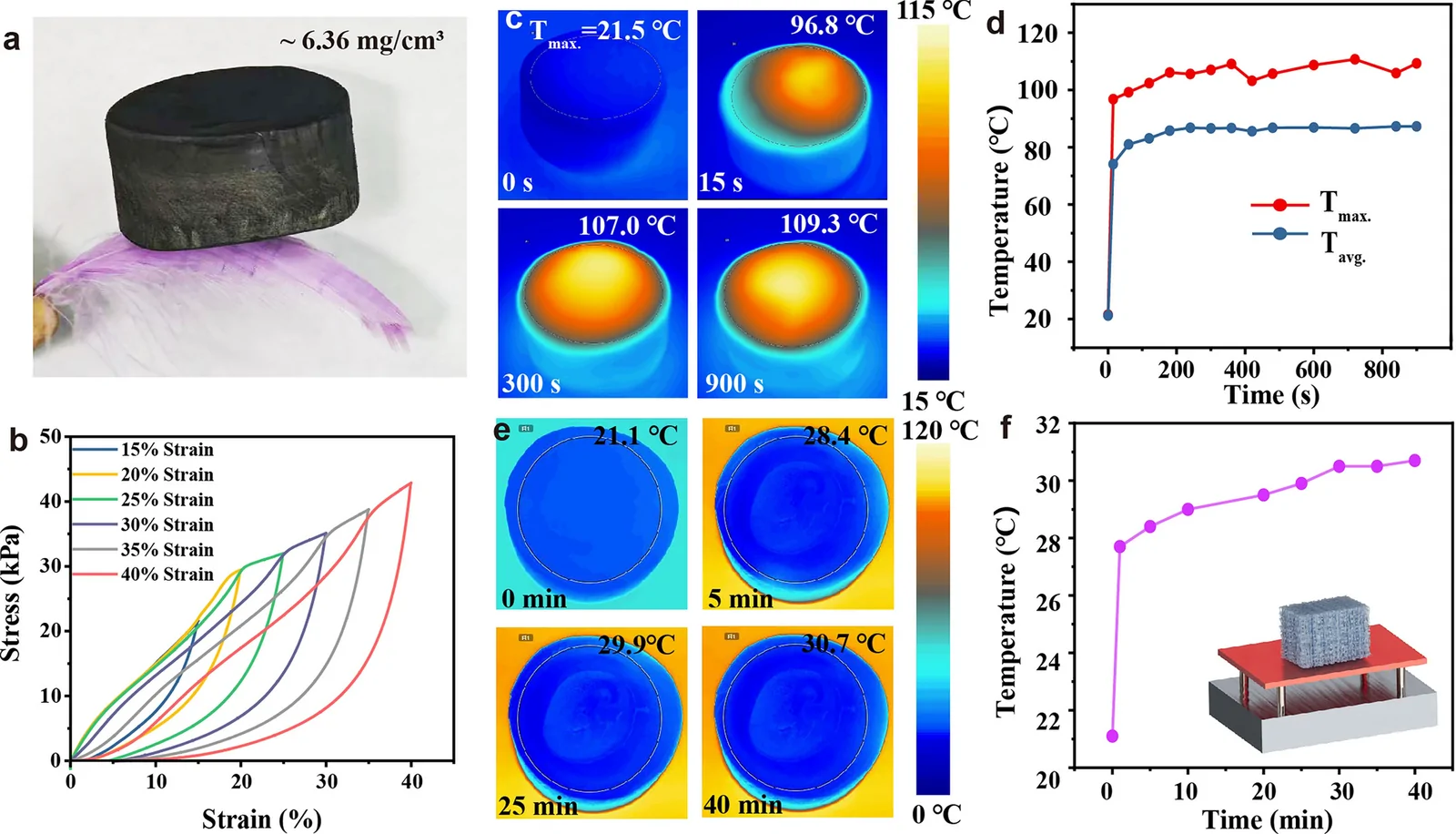
**a** Photograph of MCA-2. **b** Cyclic compressive stress–strain curves of MCA-2 at different strains. **c** Infrared thermal images and **d** temperature changes of MCA-2 under simulated sunlight irradiation (0.1 W cm^−2^). **e** Infrared thermal images of MCA-2 heating by heating plate and **f** corresponding temperature–time curves

To adapt to complex and changeable application environments, lightweight electromagnetic absorption materials are usually required with excellent mechanical and thermal management properties. As expected, the MCA-2 aerogel assembled from carbon fibers and graphene has an ultra-light density of 6.36 mg cm^−3^, enabling it to stand on a feather (Figs. 7a and S8). To explore the mechanical properties of composite aerogels, the plots of compressive stress (σ) as a function of strain (ε) for the MCA-2 aerogel at different set strains (15%–40%) are shown in Fig. 7b. The MCA-2 aerogel presents excellent reversible compressibility even under 40% strain, demonstrating its excellent structure robustness, which is of great significance for the practical applications. The GO nanosheets and TOCNF are stacked and interwoven along the growth direction of the ice crystal to form a highly oriented framework, which, together with the mechanical reinforcing effect of MCFs, gives the aerogel high strength and elasticity. Additionally, the black hybrid aerogel exhibits strong sunlight absorption, granting it exceptional photothermal conversion potential. Under simulated solar irradiation (0.1 W cm^−2^), thermal IR images and temperature profiles reveal rapid, efficient heating (Fig. 7c, d). It rapidly heats from 21 to 109 °C, demonstrating effective solar energy capture and utilization. Furthermore, after five consecutive photothermal cycles, the photothermal conversion capability of MCA-2 remains virtually unchanged, confirming excellent cycling stability (Fig. S9). To evaluate the thermal insulation properties of the aerogel, the sample was placed on a heating platform maintained at 120 °C, and its surface temperature was monitored using an infrared thermal camera. As shown in Fig. 7e, f, after 40 min of heating, the surface temperature of the aerogel remained below 31 °C, demonstrating excellent thermal stability. This performance can be attributed to the restricted heat conduction through the hierarchical porous structure, in which trapped air significantly inhibits thermal transfer. Correspondingly, the MCA‑2 aerogel exhibits a low thermal conductivity of 34.59 mW m^−1^ K^−1^ (Fig. S10), further confirming its effective insulating capability. As shown in Fig. S11, CA exhibits slightly inferior yet comparable mechanical and thermal management properties to MCA-2. This indicates that the incorporation of MCFs preserves the inherent advantages of the carbon aerogel matrix while substantially enhancing its electromagnetic wave absorption performance. Moreover, post-testing SEM characterization of MCA-2 after mechanical compression and thermal management evaluations is shown in Fig. S12. It reveals that the hierarchical porous structure remains well preserved without collapse, and the MCFs stay firmly embedded within the carbon skeleton with no signs of detachment. These observations confirm the excellent structural stability of the composite. In short, the intrinsic combination of microwave absorption with photothermal conversion and thermal insulation properties makes MCA-2 a promising candidate for dual-functional applications, particularly in complex environments requiring simultaneous electromagnetic management and thermal regulation.

## Conclusion

In summary, leveraging the advantages of lightweight and highly designable fiber and aerogel materials, we fabricated a hierarchically porous carbon aerogel loaded with MCFs via electrospinning and freeze-drying process. The integration of 1D MCFs and a 3D carbon aerogel matrix provides a solution to the drawbacks of traditional ferrite powders (high density, narrow bandwidth, high loading) as well as the impedance mismatch common to carbon materials. This multi-dimensional design endows the composite aerogel with excellent EMW absorption performance, integrating both wide bandwidth and strong absorption capability. Notably, MCA-2 exhibits an extensive EAB of 7.2 GHz with an optimal RL value of − 54.11 dB at a low filler loading of 5 wt%. Furthermore, across a thickness range of 1–5 mm, MCA-2 achieves EAB coverage over most of the C to Ku bands (4.8–18 GHz), with the corresponding average RL value reaching − 26.77 dB. Such impressive EMW absorption performance can be attributed to the modulation of the electromagnetic parameters of the entire carbon skeleton via multiple electromagnetic loss induced by MCFs. Additionally, the hierarchical porous structure and robust carbon skeleton endow the MCA-2 aerogel with light weight, structural stability, and thermal management ability, showing broad potential in multi-scenario electromagnetic protection applications. Faced with increasingly complex electromagnetic environment, the MCA composite aerogel is expected to fully realize its promise as an efficient, broadband EMW absorber. More importantly, its multi-dimensional architectural design offers a novel and scalable strategy for advancing beyond conventional powdered absorbers, paving the way for next-generation lightweight, flexible, and efficient EMW-absorbing materials.

## Supplementary Information

Below is the link to the electronic supplementary material.

Supplementary file1 (DOCX 1935 kb)
