# Supplementary material for: Entropy-Driven 1D Magnetic Carbon Fibers Embedded into 3D Aerogel Enable Broadband Electromagnetic Wave Absorption
Source: Nanomicro Lett. 2026 May 7;18:356. doi: 10.1007/s40820-026-02212-w (PMC13149682; doi:10.1007/s40820-026-02212-w)
Supplement: Supplementary file 1 — Supplementary file1 (DOCX 1935 kb) [file 40820_2026_2212_MOESM1_ESM.docx]

Supporting Information for

**Entropy-Driven 1D Magnetic Carbon Fibers Embedded into 3D Aerogel Enables Broadband Electromagnetic Wave Absorption**

Tingting Zhao ^1, 2^, Shiping Shao ^1, 2^, Ke Bi ^3,^ *, Yunxiang Tang ^1^, Lili Wu ^1, 2^, Jiurong Liu ^1, 2,^ *, Zhou Wang ^1, 2^, Fenglong Wang ^1, 2,^ *

^1^ State Key Laboratory of coatings for Advanced Equipment, Shandong University, Jinan 250061, P. R. China

^2^ School of Materials Science and Engineering, Shandong University, Jinan 250061, P. R. China

^3^ State Key Laboratory of Information Photonics and Optical Communications, School of Science, Beijing University of Posts and Telecommunications, Beijing 100876, P. R. China

*****Corresponding Author. E-mails: [bike@bupt.edu.cn](mailto:bike@bupt.edu.cn) (Ke Bi); [jrliu@sdu.edu.cn](mailto:jrliu@sdu.edu.cn) (Jiurong Liu); [fenglong.wang@sdu.edu.cn](mailto:fenglong.wang@sdu.edu.cn) (Fenglong Wang)

**S1 Experimental Section**

*Synthesis of magnetic carbon fibers (MCFs)*: The MCFs were fabricated through electrospinning process followed by a pre-oxidation and carbonization treatment. Typically, 2g of PAN powders was dispersed in 60 mL DMF after sufficiently stirring. Then Ni(acac)_2_ (5 mmol), Mn(acac)_2_ (5 mmol), Co(acac)_2_ (5 mmol), Zn(acac)_2_ (5 mmol), and Fe(acac)_3_ (40 mmol) powders were homogeneously dissolved in the above solution, thereby getting the precursor solution.

As for electrospinning process, the aforesaid solution was transferred into a glass injector with a metal needle (Inner diameter: 0.5 mm, Exposed length: 38 mm). Then the positive electrode of the high-voltage source was connected to the needle while the negative electrode was connected to the receiver covered by aluminum foil. The electrospinning parameters were systematically optimized to achieve uniform and defect-free fibers. The applied voltage was varied from 10 to 16 kV, and it was found that 13 kV yielded a stable Taylor cone and continuous jet without bead formation. When the feed rate was tested between 0.5 and 2.0 mL·h^−1^, a rate of 1.0 mL·h^−1^ provided sufficient solution supply without causing droplet formation or incomplete solvent evaporation. The receiving distance was tested between 15 and 35 cm, and 25 cm yielded the best balance of solvent evaporation and fiber collection without excessive spreading. Optimal fiber morphology was achieved at 40 °C and 50% humidity, which prevented both rapid solvent evaporation and insufficient drying. Therefore, the precursor fibers were fabricated with optimized spinning parameter settings (Positive voltage: 13 kV, Negative voltage: 2 kV, Receiving distance: 25 cm, Feed rate: 1 mL·h^−1^, Temperature: 40 ℃, Humidity: 50%) and then dried at 60 ℃ for 24 h. Afterwards, the obtained fibers were placed in a tube furnace and fully pre-oxidized in air at 260 °C for 2 h with a heating rate of 1 °C min^−1^. Finally, (Mn_0.05_Ni_0.45_Zn_0.05_Co_0.45_)Fe_2_O_4_/C MCFs were synthesized through a carbonization process (Temperature: 800 ℃, Time: 2 h, Heating rate: 5 ℃·min^−1^, Atmosphere: N_2_) and named as MCFs.

*Electromagnetic measurements*: A vector network analyzer (VNA, Agilent PNA N5244A) was used to measure the electromagnetic parameters of all aerogels in the range of 2–18 GHz *via* the coaxial line method. All the MCAs were uniformly mixed with melted paraffin and the filler content of each sample in the paraffin is 5 wt.%. And the above-mentioned mixtures were cut into toroidal rings by employing a customized metal mold (outer diameter of 7.00 mm and inner diameter of 3.04 mm). Corresponding formulas for calculating the EMW absorption performance based on the transmission line theory in the metallic backing condition were presented in Section S3.

**S2 Supplementary Figures**


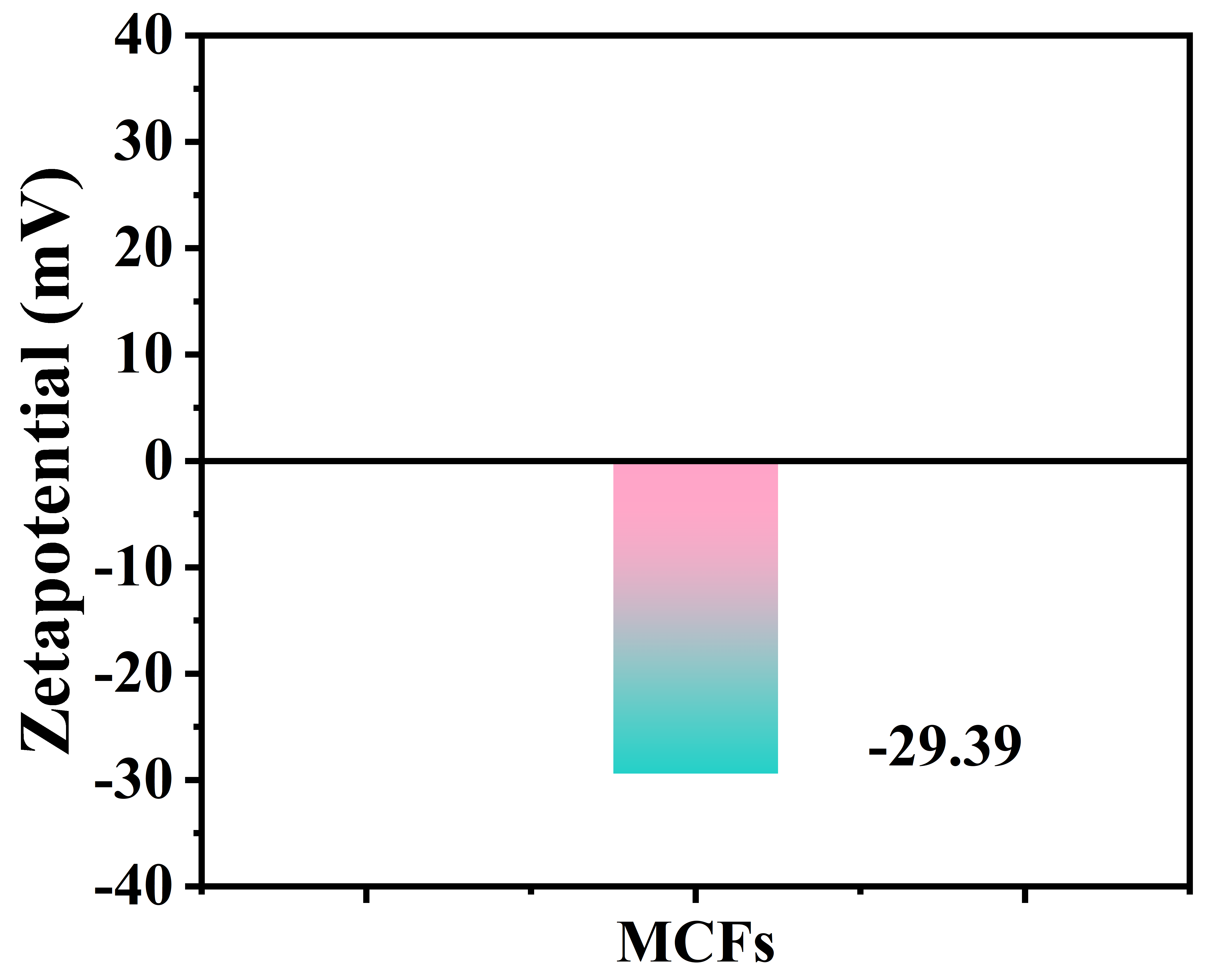


**Fig. S1** Zeta potential of MCFs


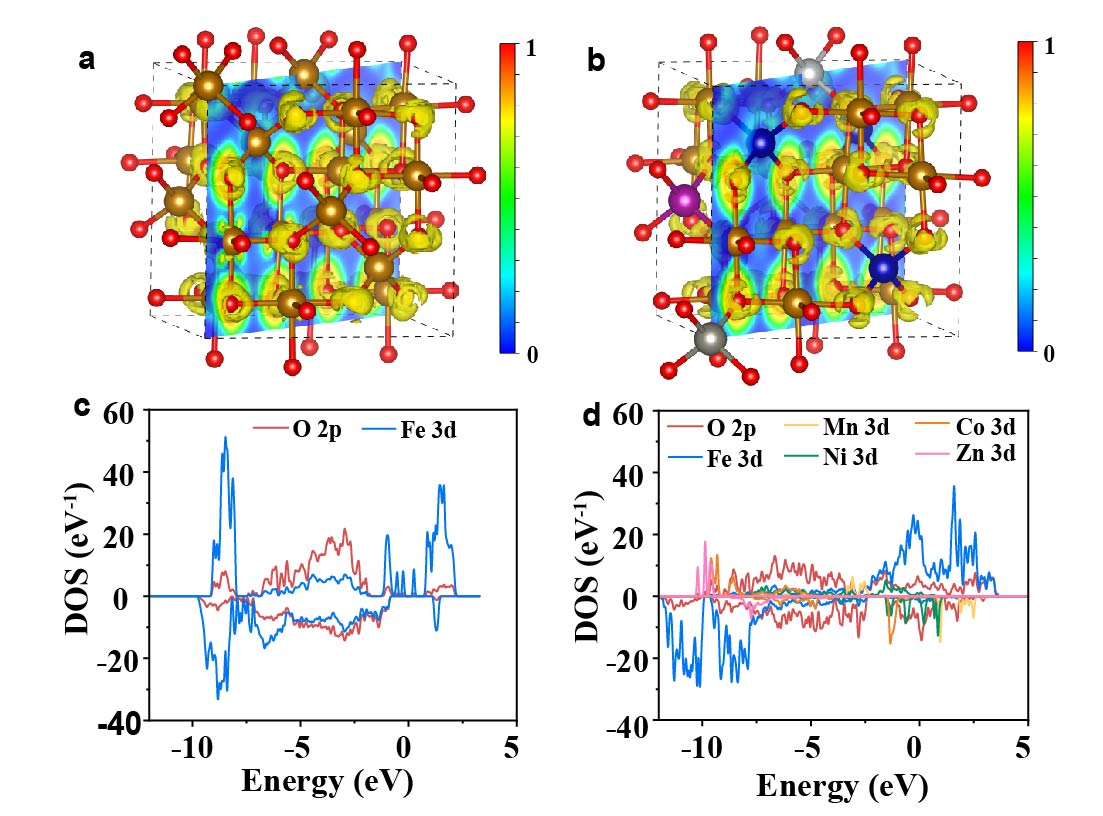


**Fig. S2** Electron localization function (ELF) of **a** Fe_3_O_4_ and **b** (Mn_0.125_Ni_0.375_Zn_0125_Co_0.375_)Fe_2_O_4_; Projected Density of States (PDOS) of **c** Fe_3_O_4_ and **d** (Mn_0.125_Ni_0.375_Zn_0.125_Co_0.375_)Fe_2_O_4_


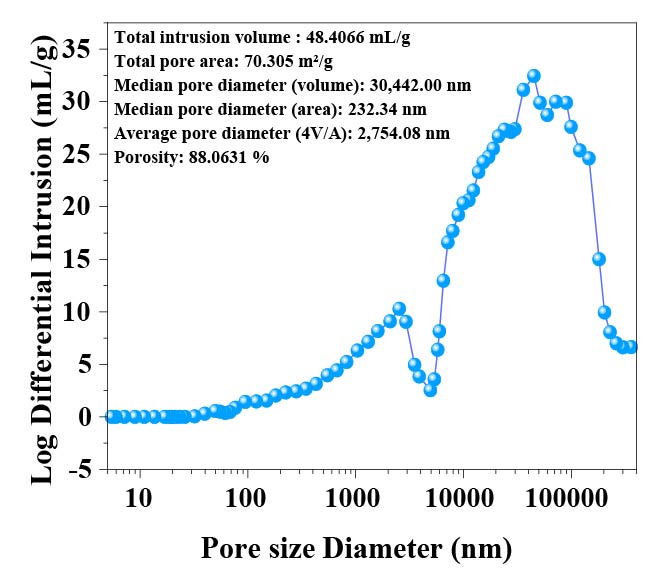


**Fig. S3** Graph of pore size distribution measured by mercury intrusion method


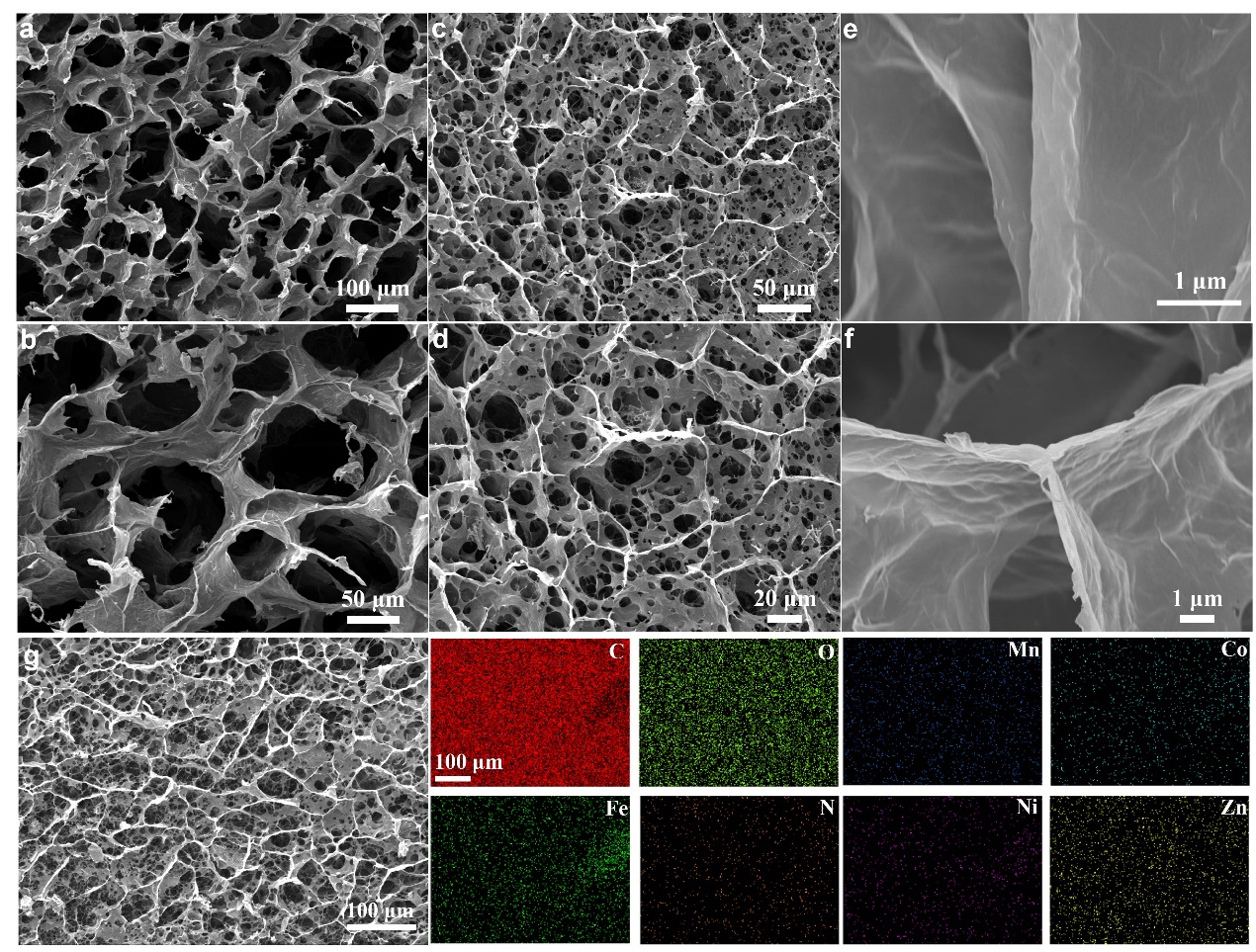


**Fig. S4 a-f** SEM images of MCA aerogels.**g** low‑magnification SEM image and corresponding EDS mapping


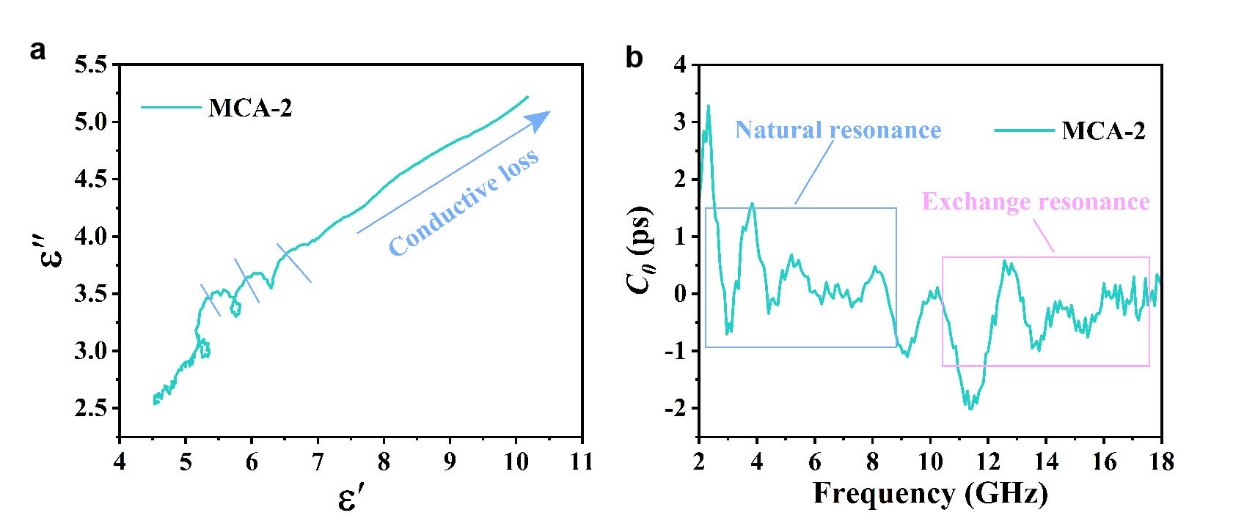


**Fig. S5 a** Cole-Cole curve and **b** *C_0_–f* curve for MCA-2


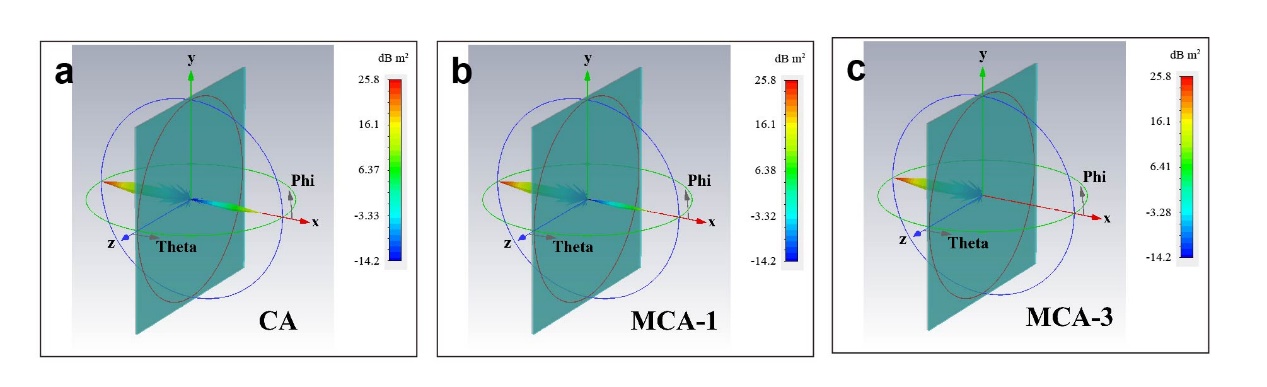


**Fig. S6** 3D RCS diagrams of **a** CA, **b** MCA-1, and **c** MCA-3


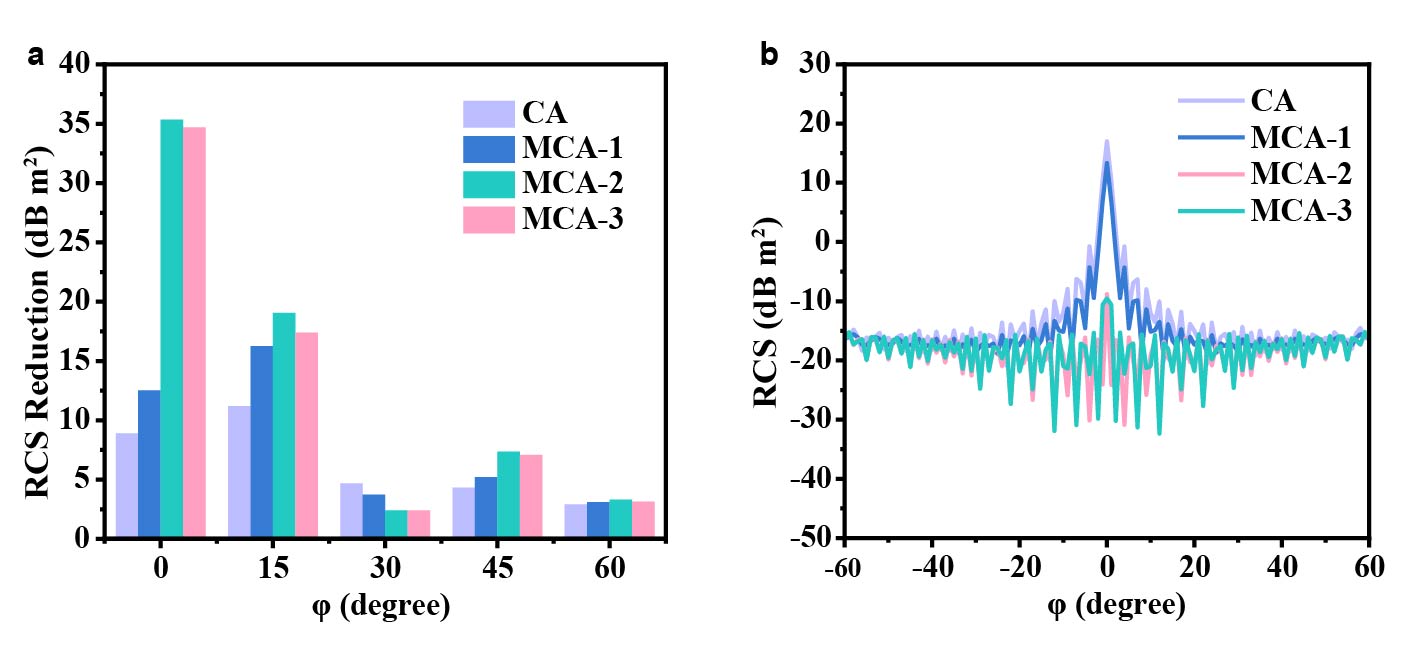


**Fig. S7** **a** RCS reduction simulation plots of PEC plane covered with MCA-2 under different detection angle. **b** 2D RCS values of the samples under horizontal polarization


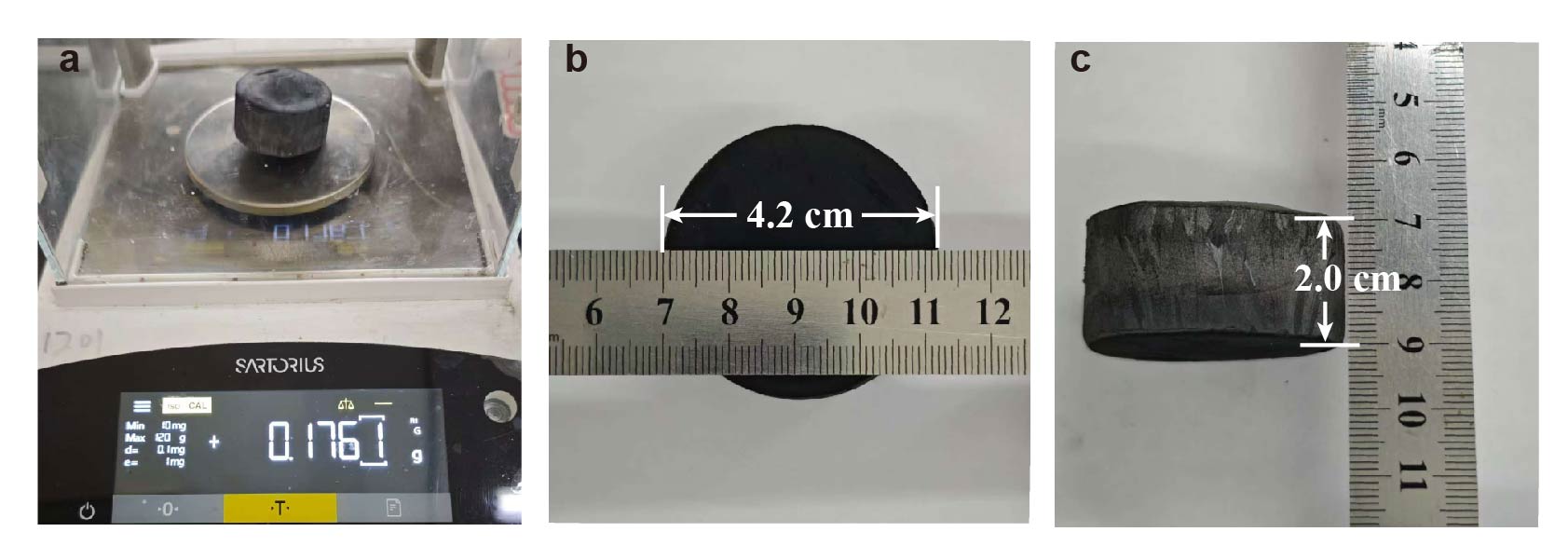


**Fig. S8** The photos of **a** mass and **b-c** size of the MCA-2 aerogel


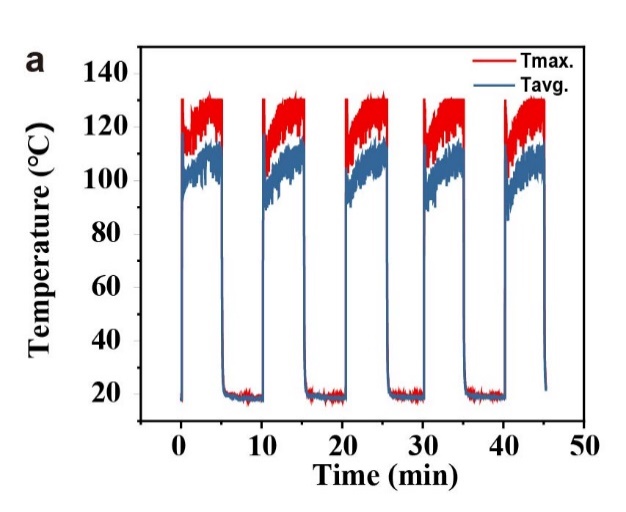


**Fig. S9** Photothermal cycling stability of MCA-2 under simulated solar irradiation (0.1 W·cm^−2^)


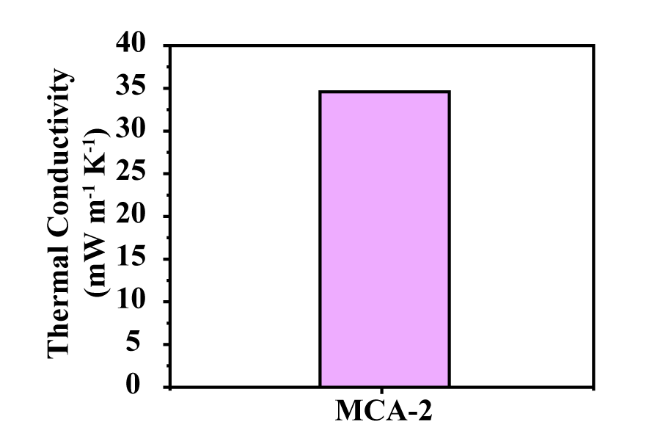


**Fig. S10** The thermal conductivity of the MCA-2 aerogel


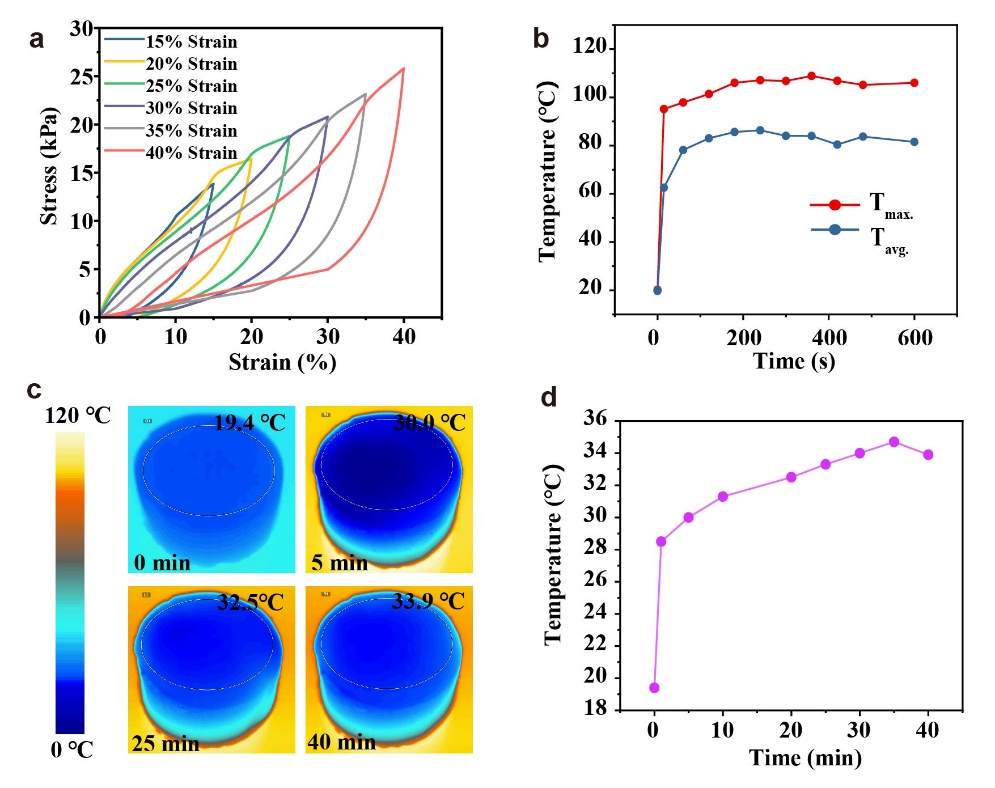


**Fig. S11 a** Cyclic compressive stress–strain curves of CA at different strains. **b** temperature changes of CA under simulated sunlight irradiation (0.1 W·cm^−2^). **c** Infrared thermal images of CA heating by heating plate and **d** corresponding temperature–time curves.


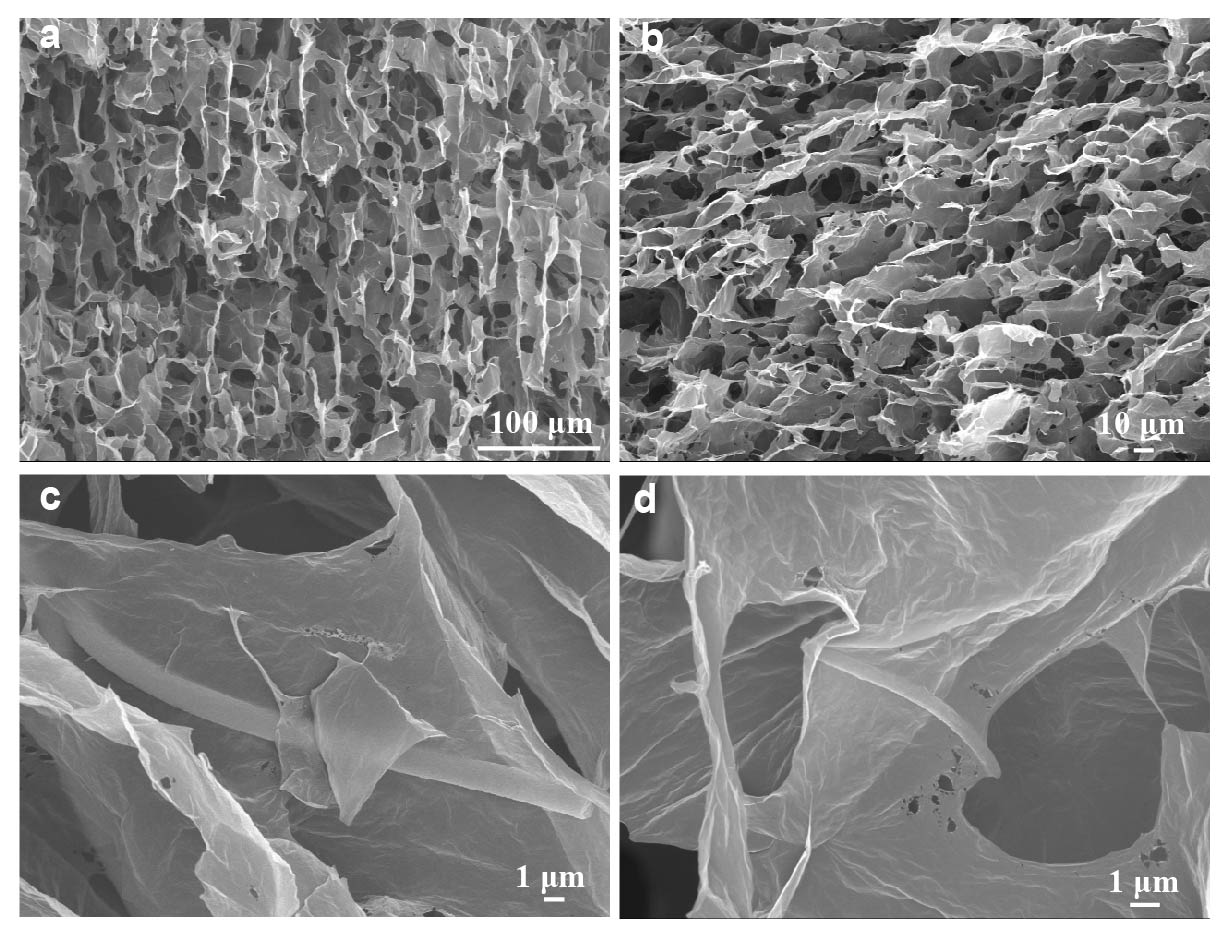


**Fig. S12 a-d S**EM images of MCA-2 after being subjected to mechanical compression and thermal performance tests, followed by 110 days of ambient storage

**S3 Supplementary Discussion**

As a reflection on the level of structural complexity or disorder, configuration entropy (*S_config_*) can be calculated as [S1, S2]:

$S_{\text{confg }}=R\ln\left[ \frac{N!}{\Pi_{i}n_{i}!} \right]\approx-R\sum_{i} x_{i}\ln x_{i}$ (**S1**)

Where *R* represents the gas constant, *N* and *x_i_* refer to the atom species in total and corresponding atomic fraction, respectively. Generally speaking, the value of *S_config_* ≥1.5 R can be regarded as high entropy, the value of *S_config_* between 1 R and 1.5 R can be considered as medium entropy and when the value of *S_config_* is below 1R, it can be deemed as low entropy.

Based on the ferromagnetic resonance theory, the natural resonance frequency can be expressed as follows [S3]:

$2\pi f_{r}=\gamma H_{\alpha}$ (**S2**)

$H_{\alpha}=4\left| K_{1} \right|/3\mu_{0}M_{s}$ (**S3**)

$K_{1}=\mu_{0}M_{s}H_{c}/2$ (**S4**)

Where *f_r_* is the natural resonance frequency, *γ* represents the gyromagnetic ratio, *H_α_* is anisotropy energy and *K*_1_ represents the anisotropy coefficient.

The calculation formula of the RL–*f* curves according to the transmission line theory in the metallic backing condition are as follows [S4]:

$\text{RL}=20\log_{10} \left| \frac{Z_{\mathrm{in}}-Z_{0}}{Z_{\mathrm{in}}+Z_{0}} \right|$ (**S5**)

$Z_{\text{in}}=Z_{0}\sqrt{\frac{\mu_{r}}{\varepsilon_{r}}}\tanh\left( \frac{2\pi jfd}{c}\sqrt{\mu_{r}\varepsilon_{r}} \right)$ (**S6**)

Together with conductive loss, the polarization-relaxation process was evaluated by the Cole–Cole curve. According to the Debye theory, the complex permittivity will conform to the following equations [S5]:

$\varepsilon^{'}=\frac{\varepsilon_{s}-\varepsilon_{\infty}}{1+\omega^{2}\tau^{2}}+\varepsilon_{\infty}$ (**S7**)

$\varepsilon^{''}=\varepsilon_{p}^{''}+\varepsilon_{c}^{''}=\omega\tau\frac{\varepsilon_{s}-\varepsilon_{\infty}}{1+\omega^{2}\tau^{2}}+\frac{\sigma}{\varepsilon_{0}\omega}$ (**S8**)

Where the *τ*, *σ* and *ω* is the polarization-relaxation time, conductivity and angular frequency, respectively, *ε_s_*, *ε_∞_* and ε_0_ represent the permittivity at the electrostatic field and high-frequency limit as well as the permittivity of vacuum, respectively.

By combining and simplifying the above equations, the following formula which shows the relationship between ε' and ε'' can be derived [S6]:

$\left( \varepsilon^{'}-\frac{\varepsilon_{s}+\varepsilon_{\infty}}{2} \right)^{2}+\left( \varepsilon^{''}-\frac{\sigma}{\varepsilon_{0}\omega} \right)^{2}=\left( \frac{\varepsilon_{s}-\varepsilon_{\infty}}{2} \right)^{2}$ (**S9**)

The following equation was utilized to recognize the action eddy current loss [S7]:

$C_{0}=\mu^{''}\left( \mu^{'} \right)^{-2}f^{-1}=2\pi\mu_{0}\sigma d^{2}/3$ (**S10**)

The attenuation ability of the EMA materials can be assessed by attenuation coefficient (*α*) as followed [S8]:

$\alpha=\frac{\sqrt{2}\pi f}{c}\times\sqrt{\left( \mu^{''}\varepsilon^{''}-\mu^{'}\varepsilon^{'} \right)+\sqrt{\left( \mu^{''}\varepsilon^{''}-\mu^{'}\varepsilon^{'} \right)^{2}+\left( \mu^{''}\varepsilon^{'}-\mu^{'}\varepsilon^{''} \right)^{2}}}$ (**S11**)

The normalized wave impedance of the samples can be an effective way to judge whether electromagnetic wave fully enter the interior of the material. It satisfies the following equation [S9]:

$Z=\left| \frac{Z_{\mathrm{in}}}{Z_{0}} \right|=\left| Z^{'}+jZ^{''} \right|$ (**S12**)

Where *Z_in_* and *Z_0_* are the input impedance of electromagnetic wave on the surface of the absorber and free space, respectively, Z′ is the real part of Z, and Z″ is the imaginary part of Z, *ε_r_* and *μ_r_* are the relative complex permittivity (*ε_r_* = *ε'*−*jε″*) and the relative complex permeability (*μ_r_* = *μ'*−*jμ″*), respectively, *d* is the thickness, *c* is the velocity of light and *f* represents the testing frequency.

**S4 Density Functional Theory (DFT) Calculations**

Spin-polarized DFT calculations were carried out to investigate the electron localization function (ELF) of the spinel ferrite particles using the Vienna ab initio Simulation Package (VASP). We employed the Perdew−Burke−Erzenhof (PBE) functional within the generalized gradient approximation (GGA) to treat the exchange-correlation energy. Structural relaxation was conducted via the conjugate gradient method, with force convergence set to a stringent criterion of 0.02 eV/Å and total energy convergence ensured until the energy difference between successive steps fell below 10^−5^ eV. The Hubbard U correction was applied to properly account for the strong on-site Coulomb interactions of transition metals.

**S5 RCS simulation**

Model construction and excitation configuration: For Fig. 6a and Fig. S6, the simulation model consists of a two-layer flat plate structure (150.0×150.0 mm^2^) comprising a perfect electric conductor (PEC) substrate (thickness: 2.0 mm) coated with an absorber layer of MCA-2 (thickness: 2.02 mm). The PEC substrate represents a typical metallic surface encountered in practical applications, where the primary objective is to minimize backscattering by coating the conductive surface with an absorbing material. The absorber thickness of 2.02 mm corresponds to the optimal matching thickness identified from experimental reflection loss measurements, ensuring that the simulation reflects the material's theoretically achievable performance. The plane wave of 18 GHz is chosen as the excitation source, corresponding to the frequency at which MCA-2 achieves its minimum reflection loss, thereby providing a best-case evaluation of its RCS reduction capability. It should be noted that the incident azimuth angles are restricted within the condition of "−60° ≤ *φ* ≤ 60°" and the polarization mode is linear. When the incident wave changes with the azimuth angle and the electric field direction is always parallel to the plate, the incident wave is called horizontal polarization wave. Otherwise, it is called vertical polarization. Thus, in horizontal polarization, the z axis is the direction of electric field. In vertical polarization, the y axis is the direction of electric field. The RCS values can be calculated as follows [S10]:

$\sigma\left( dB sm \right)=10\log\left( \left( 4\pi S/\lambda^{2} \right)\left| E_{s}/E_{i} \right|^{2} \right)$ (**S13**)

Where *E_i_* and *E_s_* represent the electric field strength of the incident and scattered waves, respectively, *S* is area of the simulated plate and *λ* is the wavelength. It should be emphasized that “*0*” value was used to replace when the imaginary part of magnetic permeability exhibits a negative value.

**S6 Supplementary Table**

**Table S1** Comparison of electromagnetic wave absorption performance of MCA-2 with recently reported representative absorbers.

| Microwave absorbing materials | EAB  (GHz) | | RL_min_  (dB) | t_RL_  (mm) | Density  (mg cm^−3^) | Filler Loading  (%) | Refs. |
| --- | --- | --- | --- | --- | --- | --- | --- |
| MCA-2 | 7.2 | | −54.11 | 2.02 | 6.36 | 5% | This  work |
| MCA-3 | 7.44 | | −39.12 | 2.07 |  | 5% |  |
| Ti_3_C_2_T_x_ MXene/PI | 6.9 | | −49.36 | 1.92 |  |  | [S11] |
| Ni/MnO-CA | 7.36 | | −64.09 | 2.53 | 53.1 | 10% | [S12] |
| MGMCA | 4.35 | | −57.5 | 1.9 | 100 |  | [S13] |
| APD MXene aerogels | 6.4 | | −45.9 | 4.7 | 37 |  | [S14] |
| Co@C/CG aerogels | 4.02 | | −45.02 | 1.5 | 17 | 30% | [S15] |
| Fe_3_O_4_@NiCo_2_O_4_/PANI/NRGO aerogels | 6 | | −41.8 | 2 |  | 50% | [S16] |
| RGO/CFs | 6.2 | | −50.58 | 2.5 | 45 | 25% | [S17] |
| Graphene/BN/SiCnws | 9.2 | | −37.8 | ~2.5 |  |  | [S18] |
| PVTMS@MWCNT nano-aerogel | 5.6 | | −36.1 | 2 |  |  | [S19] |
| Fe_3_O_4_-Fe@CNFs/Al-Fe_3_O_4_-Fe | 6.4 | | −49.5 | 2.1 |  | 30% | [S20] |
| (CrMnFeNiCu)_3_O_4_ | 4.7 | | −50.7 | 1.94 |  | 70% | [S21] |
| (CrMnFeCoZn)_3_O_4_ | 0.6 | | −14.2 | 3.89 |  | 70% | [S21] |
| (MnNiCuZn)_0.7_Co_0.3_Fe_2_O_4_/graphene | 1.1 | | −16 | 4.5 |  | 30% | [S22] |
| (Fe_0.6_Co_0.2_Ni_0.2_Cr_0.2_Mn_0.2_)_3_O_4_ | 6.1 | | −34.0 | 2.7 |  | 60% | [S23] |
| (Fe_3_CoNiCrMn)_3_O_4_ | | 3 | −35 |  |  | 60% | [S24] |

**Supplementary References**

1. B. Zhao, Z. Yan, Y. Du, L. Rao, G. Chen et al., High-entropy enhanced microwave attenuation in titanate perovskites. Adv. Mater. **35**(11), 2210243 (2023). <https://doi.org/10.1002/adma.202210243>
2. W. Zheng, G. Liang, Q. Liu, J. Li, J. A. Yuwono et al., The promise of high-entropy materials for high-performance rechargeable li-ion and na-ion batteries. Joule. **7**(12), 2732-2748 (2023). <https://doi.org/10.1016/j.joule.2023.10.016>
3. L. Liang, Q. Li, X. Yan, Y. Feng, Y. Wang et al., Multifunctional magnetic ti_3_c_2_t_x_ mxene/graphene aerogel with superior electromagnetic wave absorption performance. ACS Nano. **15**(4), 6622-6632 (2021). <https://doi.org/10.1021/acsnano.0c09982>
4. X. Zhou, H. Zhang, M. Yuan, B. Li, J. Cui et al., Dispersing magnetic nanoparticles into staggered, porous nano‐frameworks: Weaving and visualizing nanoscale magnetic flux lines for enhanced electromagnetic absorption. Adv. Funct. Mater. **35**(18), 2314541 (2024). <https://doi.org/10.1002/adfm.202314541>
5. J. Zhou, D. Lan, F. Zhang, Y. Cheng, Z. Jia et al., Self-assembled mos_2_ cladding for corrosion resistant and frequency-modulated electromagnetic wave absorption materials from x-band to ku-band. Small. **19**(52), e2304932 (2023). <https://doi.org/10.1002/smll.202304932>
6. A. Feng, D. Lan, J. Liu, G. Wu, Z. Jia. Dual strategy of a-site ion substitution and self-assembled mos_2_ wrapping to boost permittivity for reinforced microwave absorption performance. J. Mater. Sci. Technol. **180**, 1-11 (2024). <https://doi.org/10.1016/j.jmst.2023.08.060>
7. K. Li, L. Han, T. Wang, J. Zhang, J. Cheng. 4d printing mof-derived/multi-fluorination nanocomposites for ultra-efficient electromagnetic wave absorption and robust environment adaptivity. J. Mater. Chem. A. **12**(11), 6302-6317 (2024). <https://doi.org/10.1039/d3ta06898f>
8. X. Zhao, Y. Huang, X. Liu, H. Jiang, M. Yu et al., Hollow multi-layer bowknot like nanoparticles surface modified by tmds derived flexible fiber membranes for electromagnetic wave absorption. Chem. Eng. J. **483**, 149085 (2024). <https://doi.org/10.1016/j.cej.2024.149085>
9. X. Meng, J. Qiao, J. Liu, L. Wu, Z. Wang et al., Bioinspired hollow/hollow architecture with flourishing dielectric properties for efficient electromagnetic energy reclamation device. Small. **20**(11), e2307647 (2024). <https://doi.org/10.1002/smll.202307647>
10. M. Huang, L. Wang, K. Pei, B. Li, W. You et al., Heterogeneous interface engineering of bi‐metal mofs‐derived znfe_2_o_4_–zno‐fe@c microspheres via confined growth strategy toward superior electromagnetic wave absorption. Adv. Funct. Mater. **34**(3), (2023). <https://doi.org/10.1002/adfm.202308898>
11. Y. Li, C. Dong, S. Wang, P. Zhang, D. Lei et al., Insight into lightweight mxene/polyimide aerogel with high-efficient microwave absorption. Materials Today Physics. **42**, 101373 (2024). <https://doi.org/10.1016/j.mtphys.2024.101373>
12. S. Wang, X. Zhang, S. Hao, J. Qiao, Z. Wang et al., Nitrogen-doped magnetic-dielectric-carbon aerogel for high-efficiency electromagnetic wave absorption. Nano-Micro Lett. **16**(1), 16 (2023). <https://doi.org/10.1007/s40820-023-01244-w>
13. M. Shen, J. Qi, X. Xu, J. Li, Y. Xu et al., Promoting electromagnetic wave absorption performance by integrating mos_2_@gd_2_o_3_/mxene multiple hetero-interfaces in wood-derived carbon aerogels. Small. **20**(12), e2306915 (2024). <https://doi.org/10.1002/smll.202306915>
14. Y. Sui, N. Wu, Y. Liu, J. Lin, Y. Deng et al., Hydrophobic interaction-directed solvent-free ambient-pressure-dried mxene aerogels. Adv. Mater. **38**(11), e14667 (2026). <https://doi.org/10.1002/adma.202514667>
15. J. Xu, X. Zhang, Z. Zhao, H. Hu, B. Li et al., Lightweight, fire-retardant, and anti-compressed honeycombed-like carbon aerogels for thermal management and high-efficiency electromagnetic absorbing properties. Small. **17**(33), e2102032 (2021). <https://doi.org/10.1002/smll.202102032>
16. Y. Mehdizadeh, J. Seyed-Yazdi, F. Jamadi, F. Ebrahimi-Tazangi, S. J. Seyyedyazdi et al., Ultra-broadband microwave absorption performance of multi-scale designed fe_3_o_4_@nico_2_o_4_/pani/nrgo aerogels. J. Mater. Chem. A. **13**(29), 23766-23785 (2025). <https://doi.org/10.1039/d5ta03371c>
17. B. Zhan, Y. Qu, X. Qi, J. Ding, J. J. Shao et al., Mixed-dimensional assembly strategy to construct reduced graphene oxide/carbon foams heterostructures for microwave absorption, anti-corrosion and thermal insulation. Nano-Micro Lett. **16**(1), 221 (2024). <https://doi.org/10.1007/s40820-024-01447-9>
18. X. You, H. Ouyang, R. Deng, Q. Zhang, Z. Xing et al., Graphene aerogel composites with self-organized nanowires-packed honeycomb structure for highly efficient electromagnetic wave absorption. Nano-Micro Lett. **17**(1), 47 (2024). <https://doi.org/10.1007/s40820-024-01541-y>
19. H. Ma, M. Fashandi, Z. B. Rejeb, X. Ming, Y. Liu et al., Efficient electromagnetic wave absorption and thermal infrared stealth in pvtms@mwcnt nano-aerogel via abundant nano-sized cavities and attenuation interfaces. Nano-Micro Lett. **16**(1), 20 (2023). <https://doi.org/10.1007/s40820-023-01218-y>
20. X. Liu, J. Zhou, Y. Xue, X. Lu. Structural engineering of hierarchical magnetic/carbon nanocomposites via in situ growth for high-efficient electromagnetic wave absorption. Nano-Micro Lett. **16**(1), 174 (2024). <https://doi.org/10.1007/s40820-024-01396-3>
21. G. Zhang, R. Zhang, Z. Zeng, B. Liu, Y. Hou et al., Electron delocalization engineering on 2d high‐entropy metal oxides for boosting electromagnetic wave absorption. Adv. Funct. Mater. e27135 (2025). <https://doi.org/10.1002/adfm.202527135>
22. F. Hosseini Mohammadabadi, S. M. Masoudpanah, S. Alamolhoda, H. R. Koohdar. Electromagnetic microwave absorption properties of high entropy spinel ferrite ((mnnicuzn)_1−x_co_x_fe_2_o_4_)/graphene nanocomposites. Journal of Materials Research and Technology. **14**, 1099-1111 (2021). <https://doi.org/10.1016/j.jmrt.2021.07.018>
23. G. Dai, R. Deng, T. Zhang, Y. Yu, L. Song. Quantitative evaluation of loss capability for in situ conductive phase enhanced microwave absorption of high‐entropy transition metal oxides. Adv. Funct. Mater. **32**(35), 2205325 (2022). <https://doi.org/10.1002/adfm.202205325>
24. G. Dai, R. Deng, X. You, T. Zhang, Y. Yu et al., Entropy-driven phase regulation of high-entropy transition metal oxide and its enhanced high-temperature microwave absorption by in-situ dual phases. J. Mater. Sci. Technol. **116**, 11-21 (2022). <https://doi.org/10.1016/j.jmst.2021.11.032>
